# Supplementary material for: Paralemnolins X and Y, New Antimicrobial Sesquiterpenoids from the Soft Coral Paralemnalia thyrsoide
Source: Antibiotics (Basel). 2021 Sep 24;10(10):1158. doi: 10.3390/antibiotics10101158 (PMC8532672; doi:10.3390/antibiotics10101158)
Supplement: Supplementary file 1 [file antibiotics-10-01158-s001.zip › antibiotics-1391151-supplementary.pdf]

Supplementary material

Paralemnolins X and Y, New Antimicrobial Sesquiterpenoids from the Soft Coral *Paralemnalia thyrsoide*

Abdelsamed I. Elshamy[1], Tarik A. Mohamed[2], Eman M. Elkady[3], Ibrahim A. Saleh[2], Ahmed A. El-Beih[4], Montaser A. Alhammady[5], Shinji Ohta[6], Akemi Umeyama[7], Paul W. Pare[8], Mohamed-Elamir F. Hegazy[2,9]

[1]Department of Natural Compounds Chemistry, National Research Centre, 33 El-Bohouth St., Dokki, Giza 12622, Egypt; ai.el-shamy@nrc.sci.eg (A.I.E.).

[2]Chemistry of Medicinal Plants Dept., National Research Centre, 33 El-Bohouth St., Dokki, Giza 12622, Egypt; ta.mourad@nrc.sci.eg (T.A.M.); ia.saleh@ nrc.sci.eg (I.A.S.).

[3]National Institute of Oceanography & Fisheries, NIOF, Cairo 11516, Egypt; emelkady@yahoo.com (E.M.E.); coralreef_noif1@yahoo.com (M.A.A.).

[4]Chemistry of Natural & Microbial Products Dept., National Research Centre, Dokki, Giza 12622, Egypt; aa.el-beih@nrc.sci.eg (A.A.E.).

[5]National Institute of Oceanography and Fisheries, Red Sea Branch, Hurghada 84511, Egypt; E-Mail: cor-alreef_niof1@yahoo.com (M.A.A.).

[6]Graduate School of Integrated Sciences for Life, Hiroshima University, 1-7-1 Kagamiyama, Higashi- Hiroshima 739-8521, Japan; ohta@hiroshima-u.ac.jp (S.O.).

[7]Faculty of Pharmaceutical Sciences, Tokushima Bunri University, Yamashiro-cho, Tokushima, 770-8514, Japan; umeyama@ph.bunri-u.ac.jp (A.U.)

[8]Department of Chemistry & Biochemistry Texas Tech University, Lubbock, TX 79409 USA; paul.pare@ttu.edu (P.W.P.).

[9]Department of Pharmaceutical Biology, Institute of Pharmaceutical and Biomedical Sciences, Johannes Guten-berg University, Staudinger Weg 5, 55128 Mainz, Germany.

* Correspondence: paul.pare@ttu.edu (P.W.P.); mohegazy@uni-mainz.de (M.E.F.H.); Tel.: +1-806-834-0461(P.W.P); Tel.: +2 033 371 635 (M.E.F.H.)

Supporting data	Page
S1: LRCIMS of **1** …….…………………………………………………...	4
S2: HRCIMS of **1** ………………………………………………...	4
S3: ^{1}H NMR of **1** ……..…………………………………………..	5
S4:^{13}C NMR of **1** …………………………………………………….	6
S5: DEPT 135 of **1** ………………………………………………...	7
S6: HSQC of **1** ………………………………………………………..	8
S7: HMBC of **1**………………………………………………….	9
S8: ^{1}H ^{1}H COSY of **1**……………….………………………………	10
S9: NOESY of **1** …………………………………………………...	11
S10: LRCIMS of **2** …..…………………………………………...	12
S11: HRCIMS of **2** ………………………………………………...	13
S12: ^{1}H NMR of **2** ……..…………………………………………..	13
S13:^{13}C NMR of **2** ……………………………………………….	14
S14: DEPT 135 of **2** ………………………………………………...	15
S15: HSQC of **2** ………………………………………………………..	16
S16: HMBC of **2**………………………………………………….	17
S17: ^{1}H ^{1}H COSY of **2**……………….………………………………	18
S18: NOESY of **2** …………………………………………………...	19
S19: LRCIMS of **3** …………………………………………………...	20
S20: ^{1}H NMR of **3** ……..…………………………………………..	21
S21:^{13}C NMR of **3** ……………………………………………….	22
S22: LRCIMS of **4** …………………………………………………...	23
S23: ^{1}H NMR of **4** ……..…………………………………………..	24
S24:^{13}C NMR of **4** ……………………………………………….	25
S25: LRCIMS of **5** …………………………………………………...	26
S26: ^{1}H NMR of **5** ……..…………………………………………..	27
S27:^{13}C NMR of **5** ……………………………………………….	28
S28: LRCIMS of **6** …………………………………………………...	29
S29: ^{1}H NMR of **6** ……..…………………………………………..	30
S30:^{13}C NMR of **6** ……………………………………………….	31
S31: LRCIMS of **7** …………………………………………………...	32
S32: ^{1}H NMR of **7** ……..…………………………………………..	33

S33: ^{13}C NMR of **7** ………………………………………………………	34
S34: LRCIMS of **8** ……………………………………………………...	35
S35: ^{1}H NMR of **8** …….………………………………………………..	36
S36: ^{13}C NMR of **8** ………………………………………………………	37
S37: LRCIMS of **9** ……………………………………………………...	38
S38: ^{1}H NMR of **9** …….………………………………………………..	39
S39: ^{13}C NMR of **9** ………………………………………………………	40
S40: LRCIMS of **10** …………………………………………………...	41
S41: ^{1}H NMR of **10**…..……………………………………………..	42
S42: ^{13}C NMR of **10** …………………………………………………….	43
S43: LRCIMS of **11** …………………………………………………...	44
S44: ^{1}H NMR of **11** ………………………………………………..	45
S45: ^{13}C NMR of **11** …………………………………………………….	46
S46: LRCIMS of **12** …………………………………………………...	47
S47: ^{1}H NMR of **12** …….……………………………………………..	48
S48: ^{13}C NMR of **12** …………………………………………………….	49

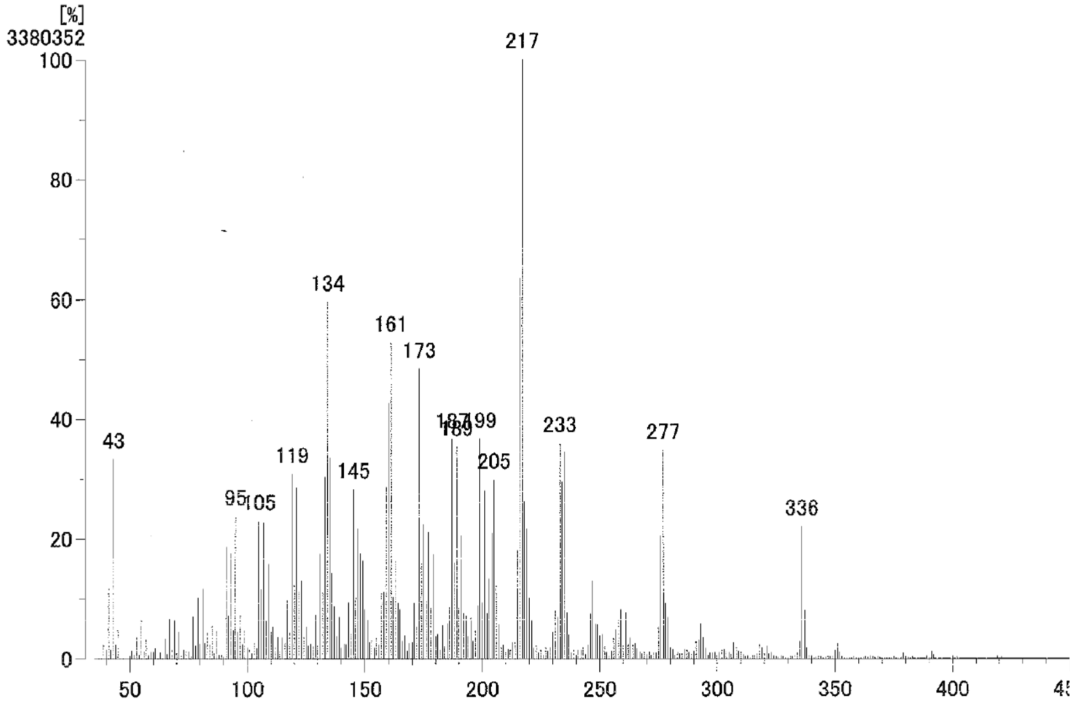

S1: LRCIMS of **1**

Data : Umeyama-CIHR.06-Aug-2019.001 Date : 06-Aug-2019 15:54
Instrument : MStation
Sample : PT-312
Note : MStation
Inlet : Direct Ion Mode : CI+
RT : 0.56 min Scan# : 15
Elements : C 150/0, H 250/0, O 50/0
Mass Tolerance : 5mmu
Unsaturation (U.S.) : 0.0 - 15.0

	Observed m/z	Int%	Err[ppm / mmu]	U.S.	Composition
1	336.1940	100.00	+1.0 / +0.3	6.0	C19 H28 O5

S2: HRCIMS of **1**

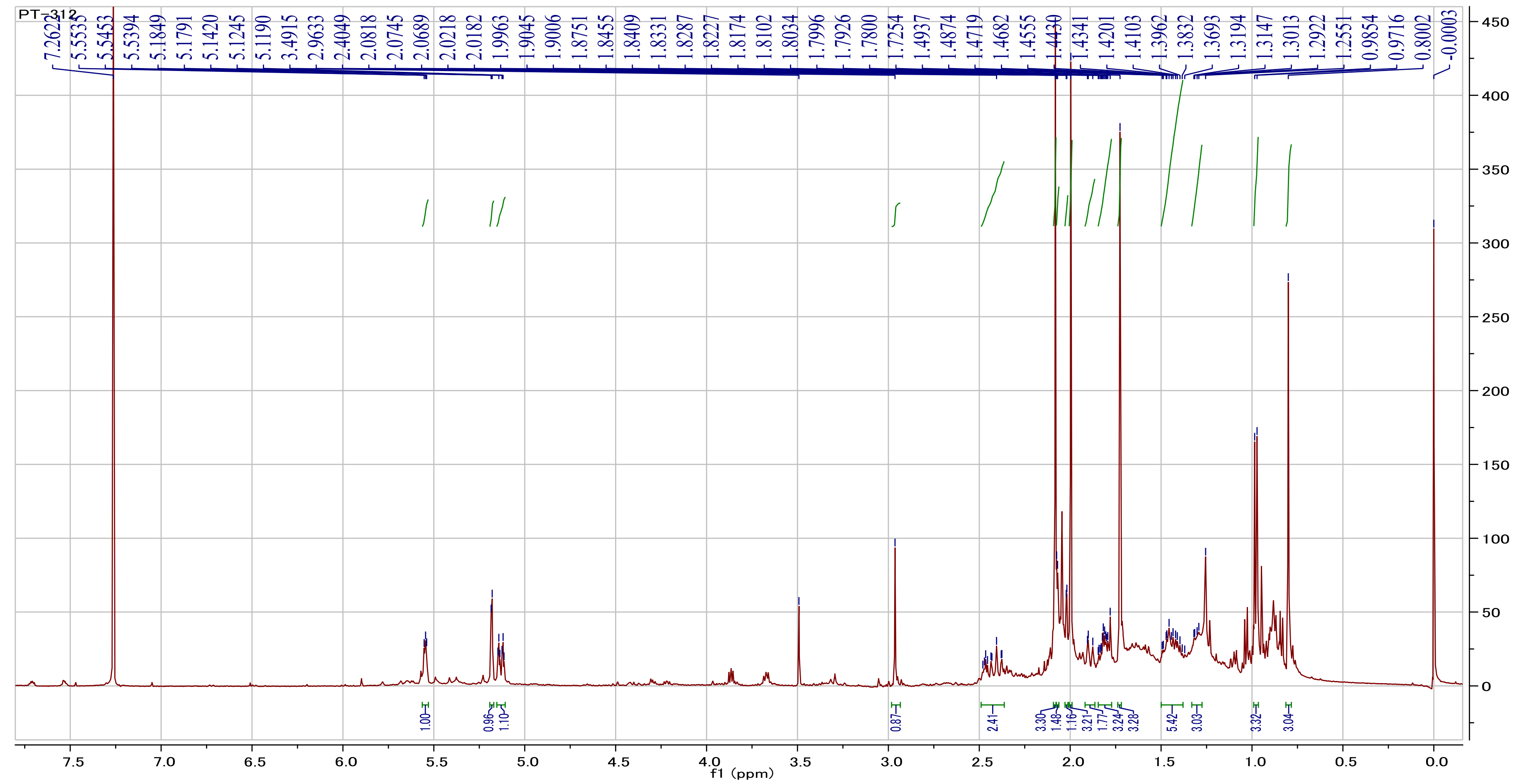

S3: ^{1}H NMR of **1**

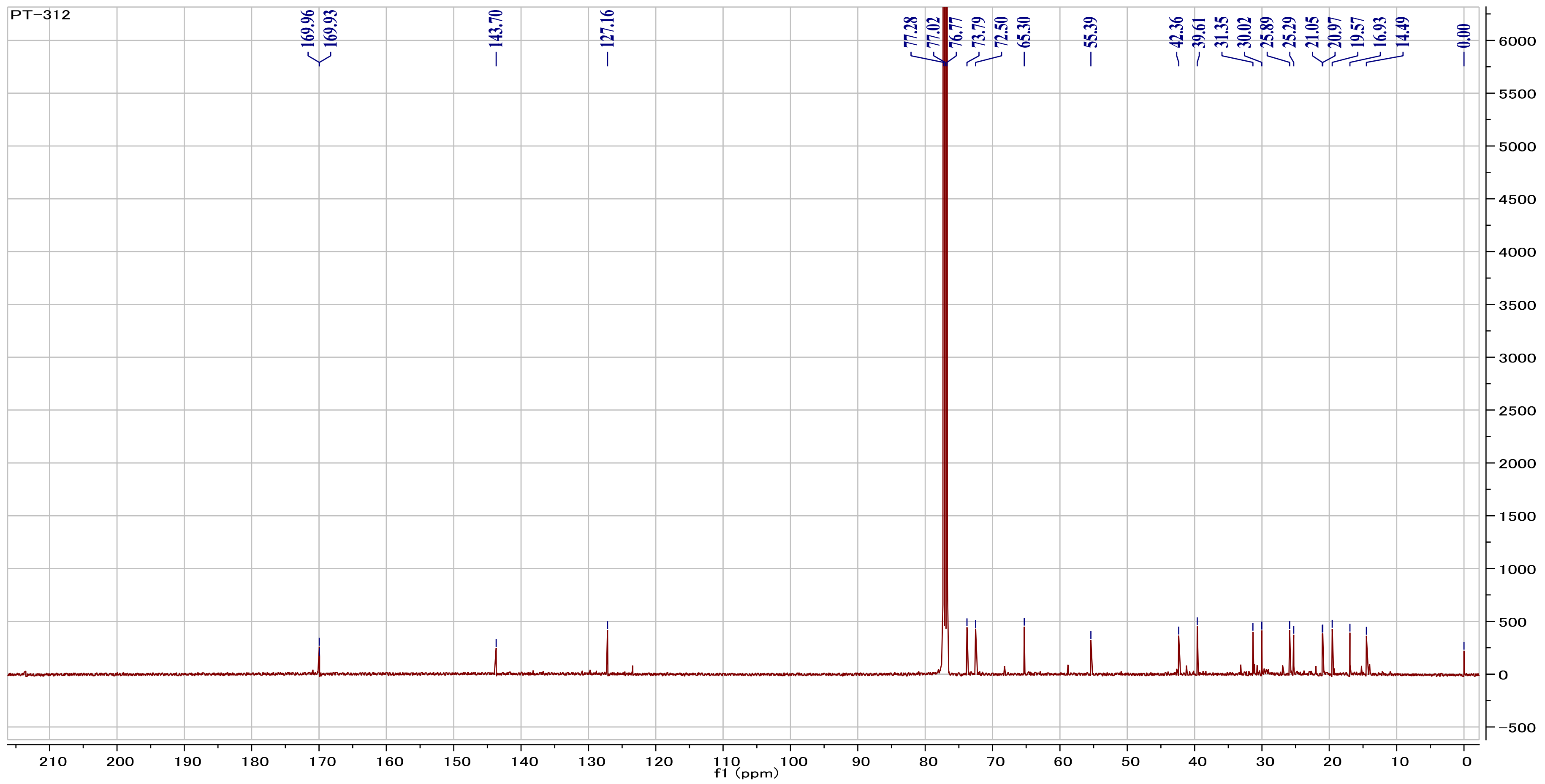

S4: ^{13}C NMR of **1**

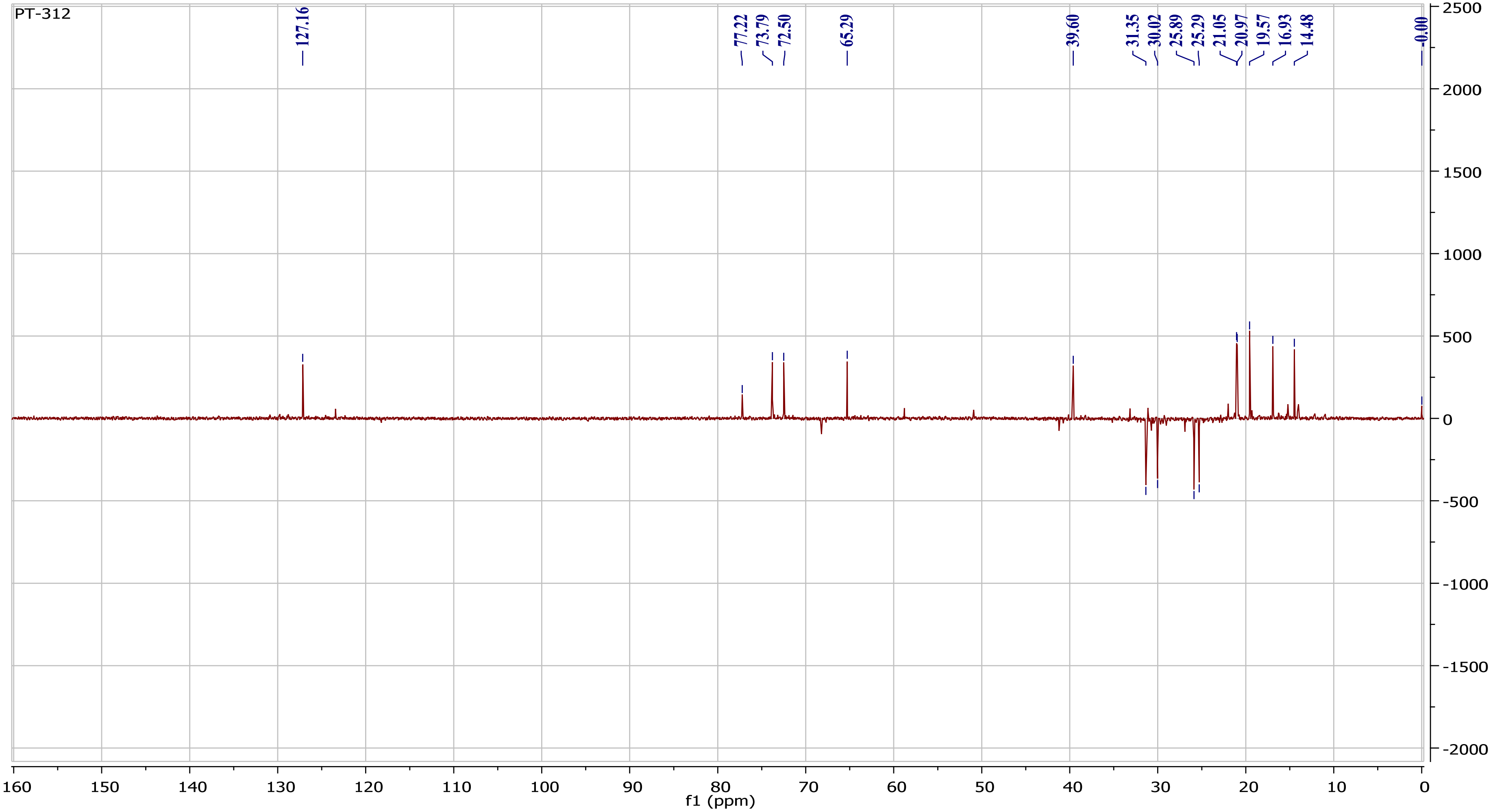

S5: DEPT-135 of **1**

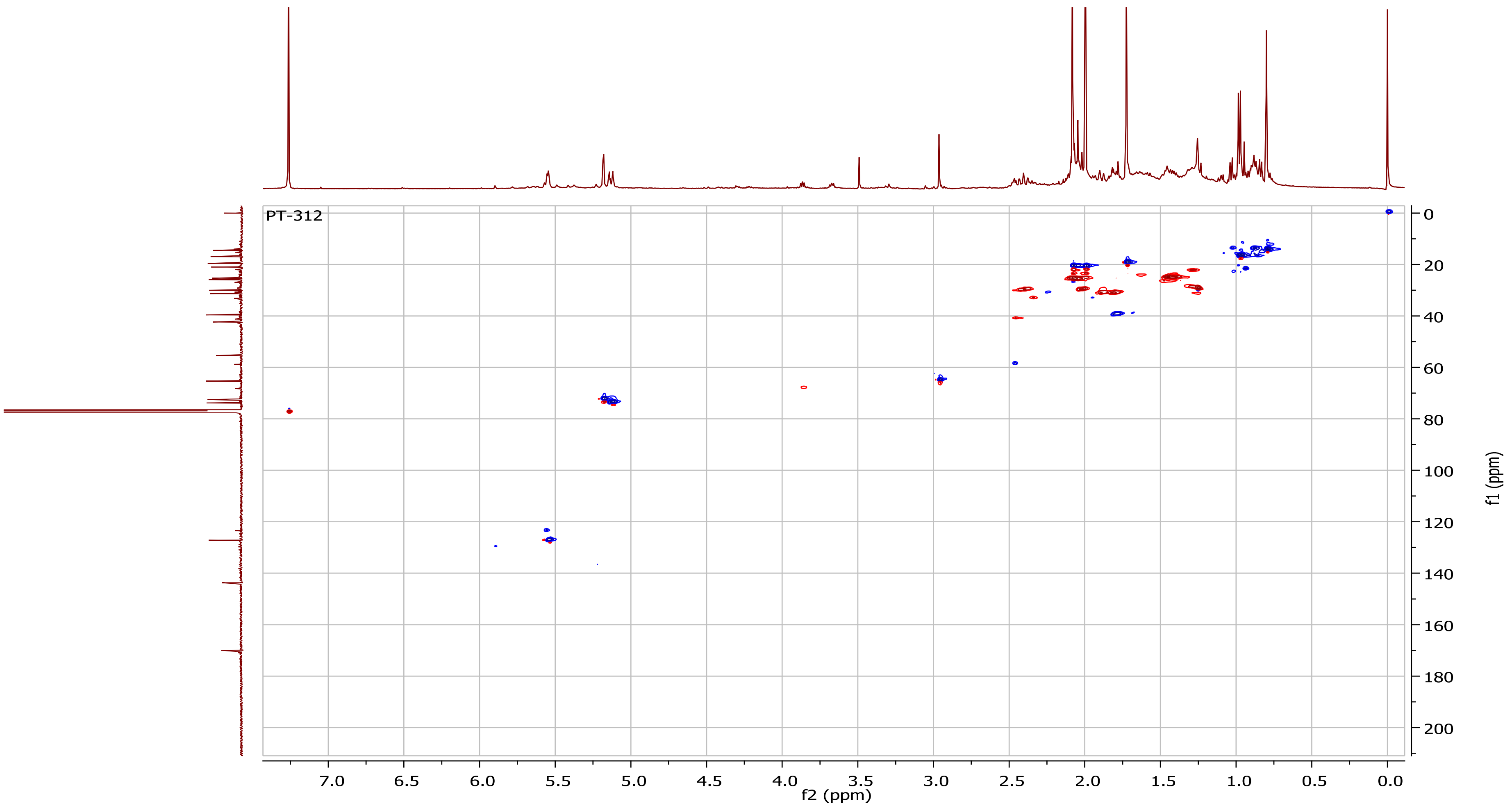
PT-312
f2 (ppm)
f1 (ppm)
7.0
6.5
6.0
5.5
5.0
4.5
4.0
3.5
3.0
2.5
2.0
1.5
1.0
0.5
0.0
0
20
40
60
80
100
120
140
160
180
200

S6: HSQC of **1**

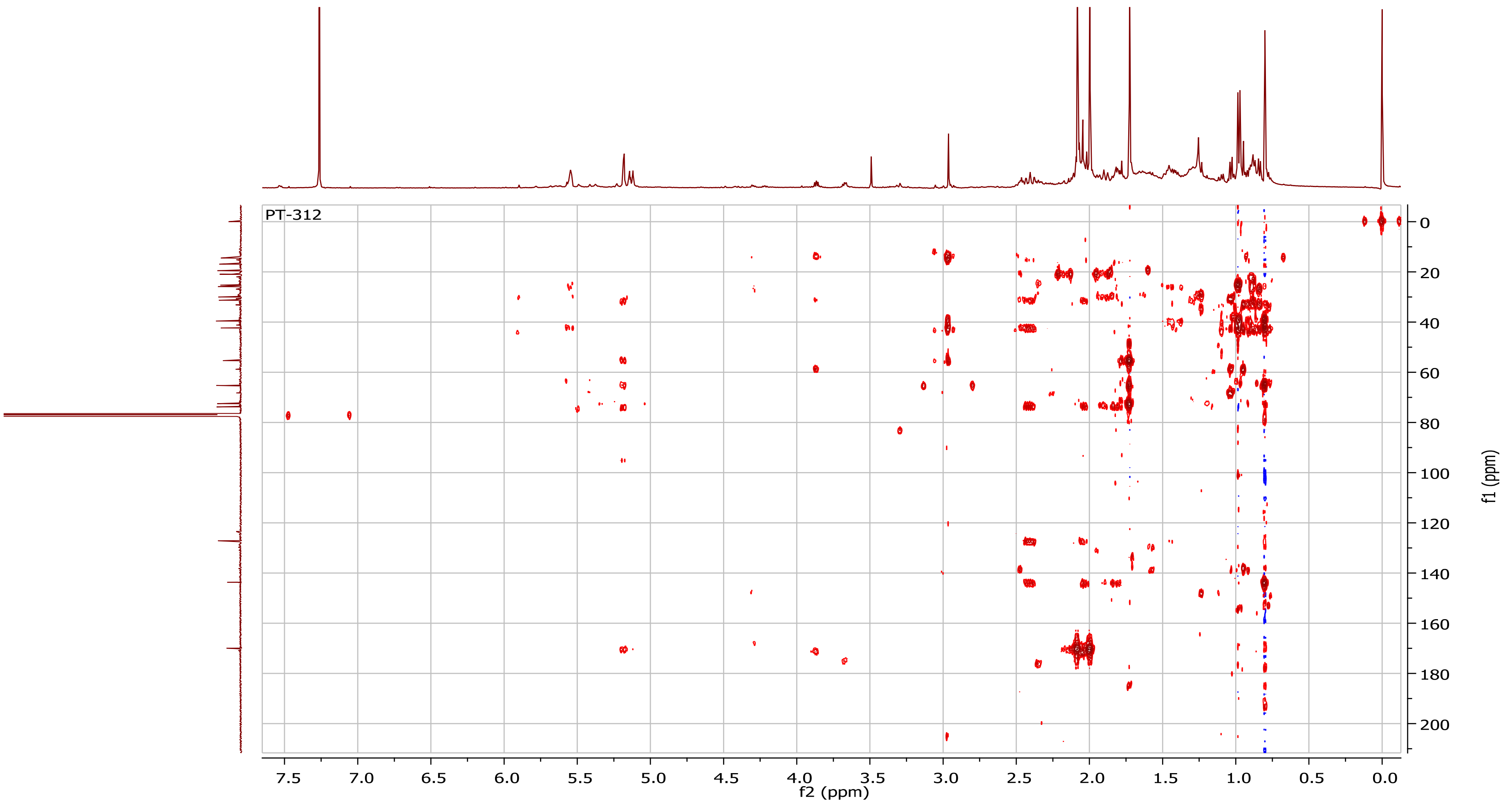

S7: HMBC of **1**

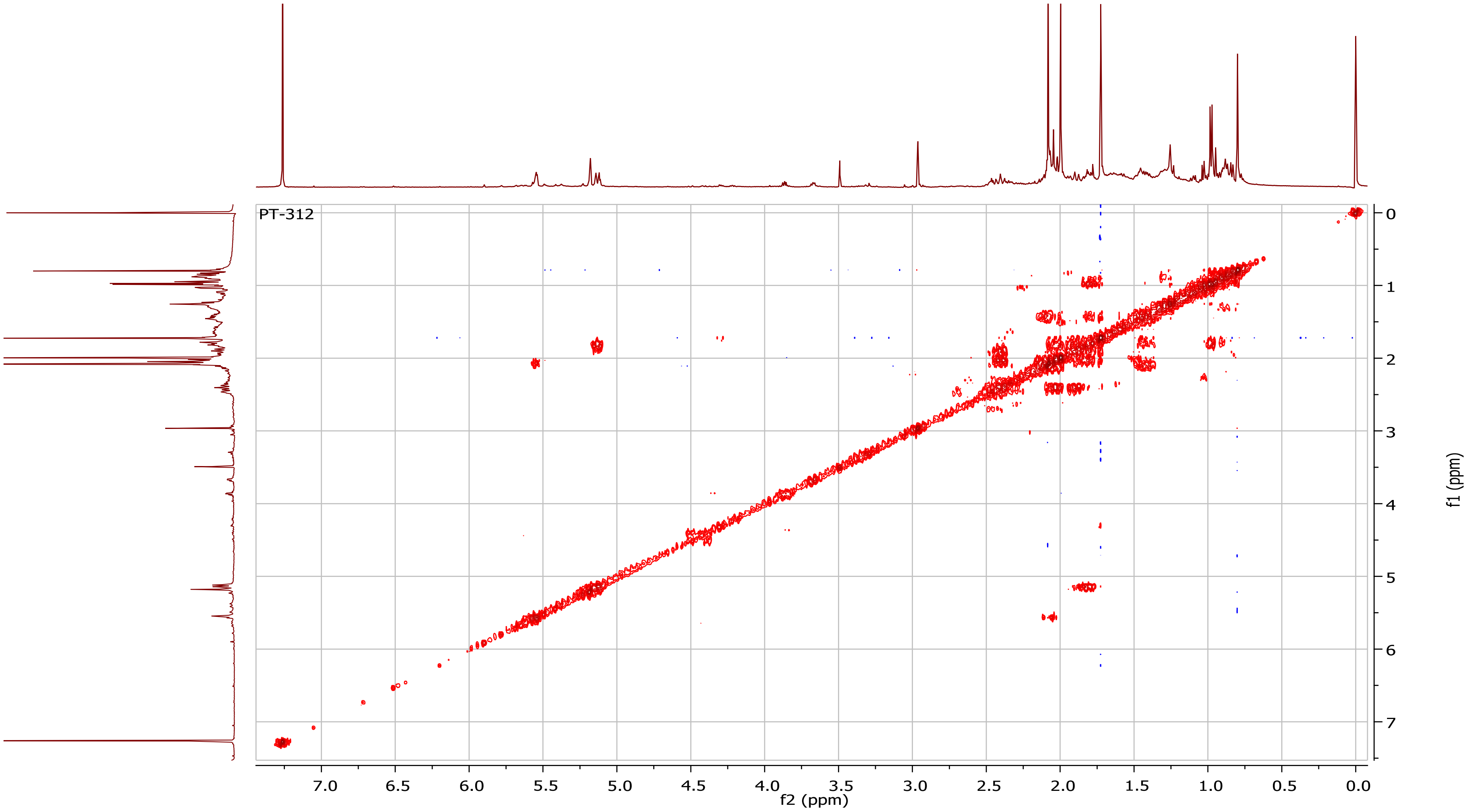

S8: ^{1}H ^{1}H COSY of **1**

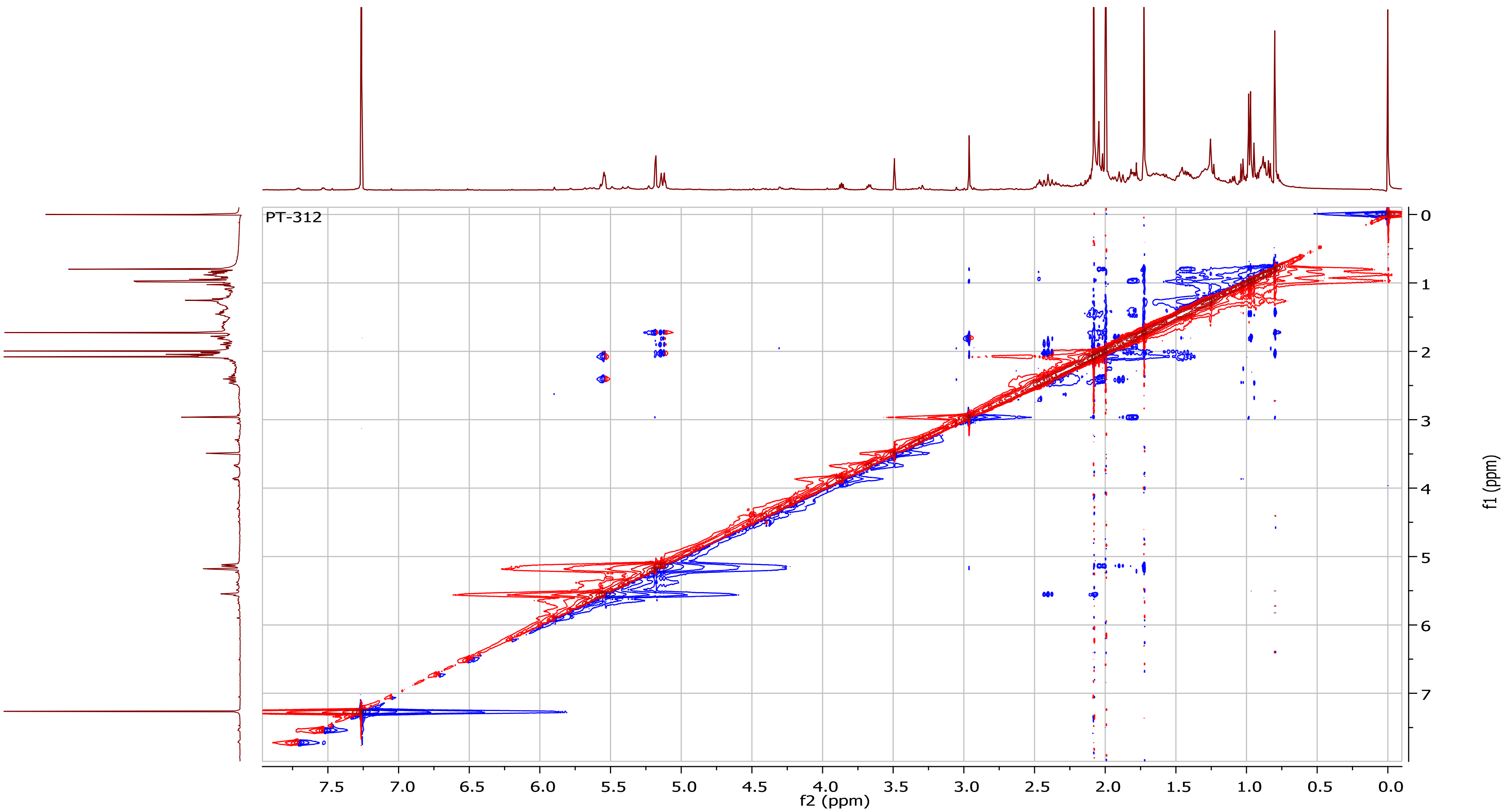

S9: NOESY of **1**

LRCIMS

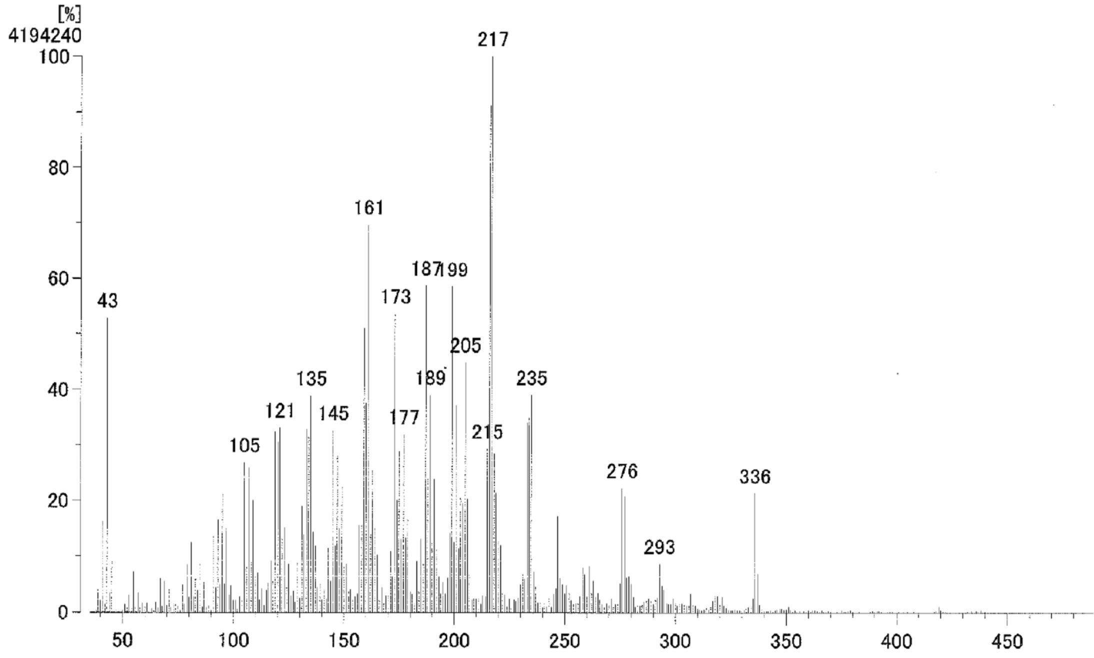

S10: LRCIMS of **2**

Data : Umeyama-CIHR.10-Aug-2019.001   Date : 10-Aug-2019 09:51
Instrument : MStation
Sample : PT-313
Note : MStation
Inlet : Direct   Ion Mode : CI+
RT : 0.40 min   Scan# : 11
Elements : C 150/0, H 250/0, O 50/0
Mass Tolerance : 5mmu
Unsaturation (U.S.) : 0.0 - 15.0

	Observed m/z	Int%	Err[ppm / mmu]	U.S.	Composition
1	336.1934	21.76	-0.8 / -0.3	6.0	C19 H28 O5

S11: HRCIMS of **2**

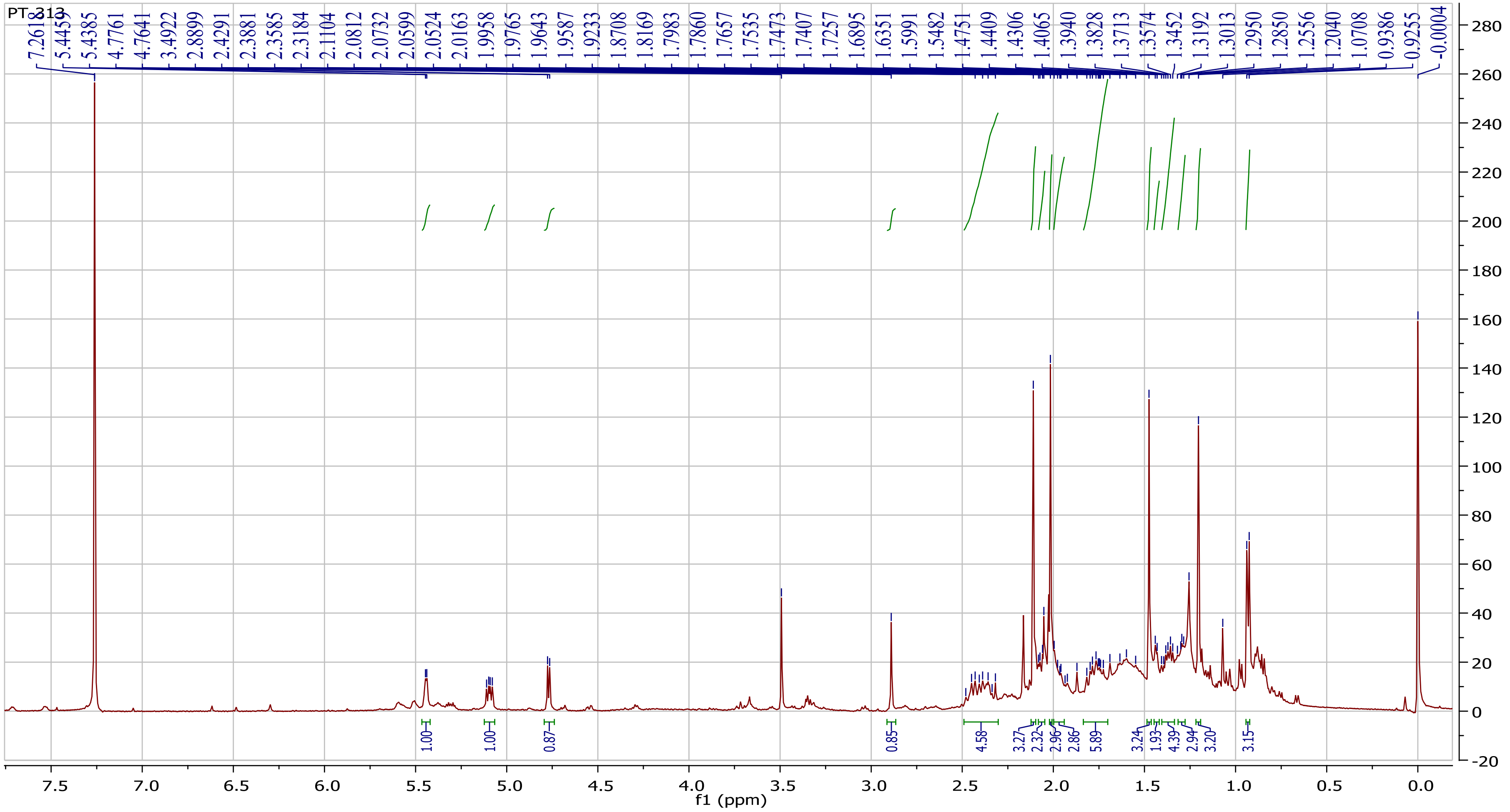

S12: ^{1}H NMR of **2**

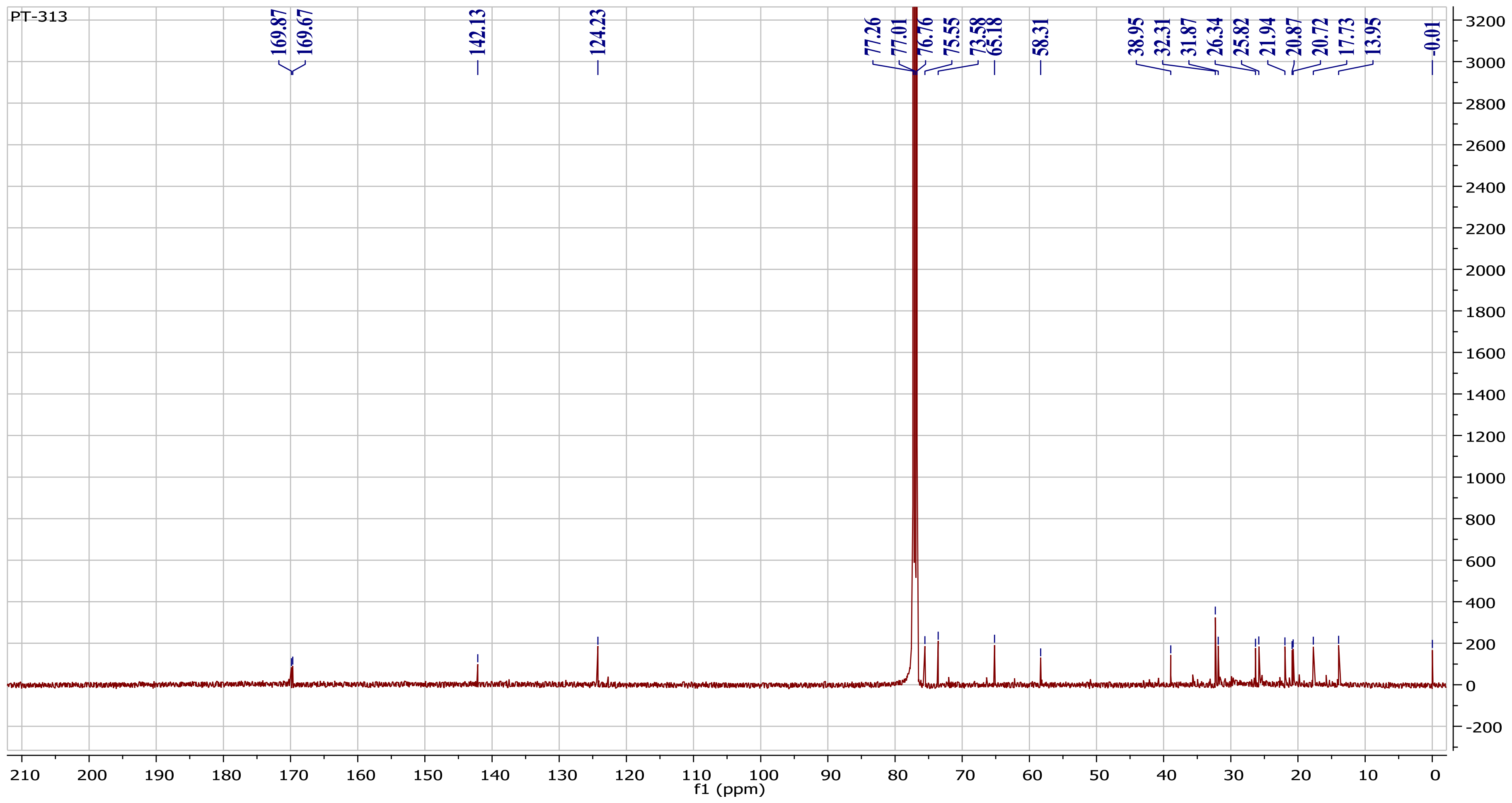

S13:^{13}C NMR of **2**

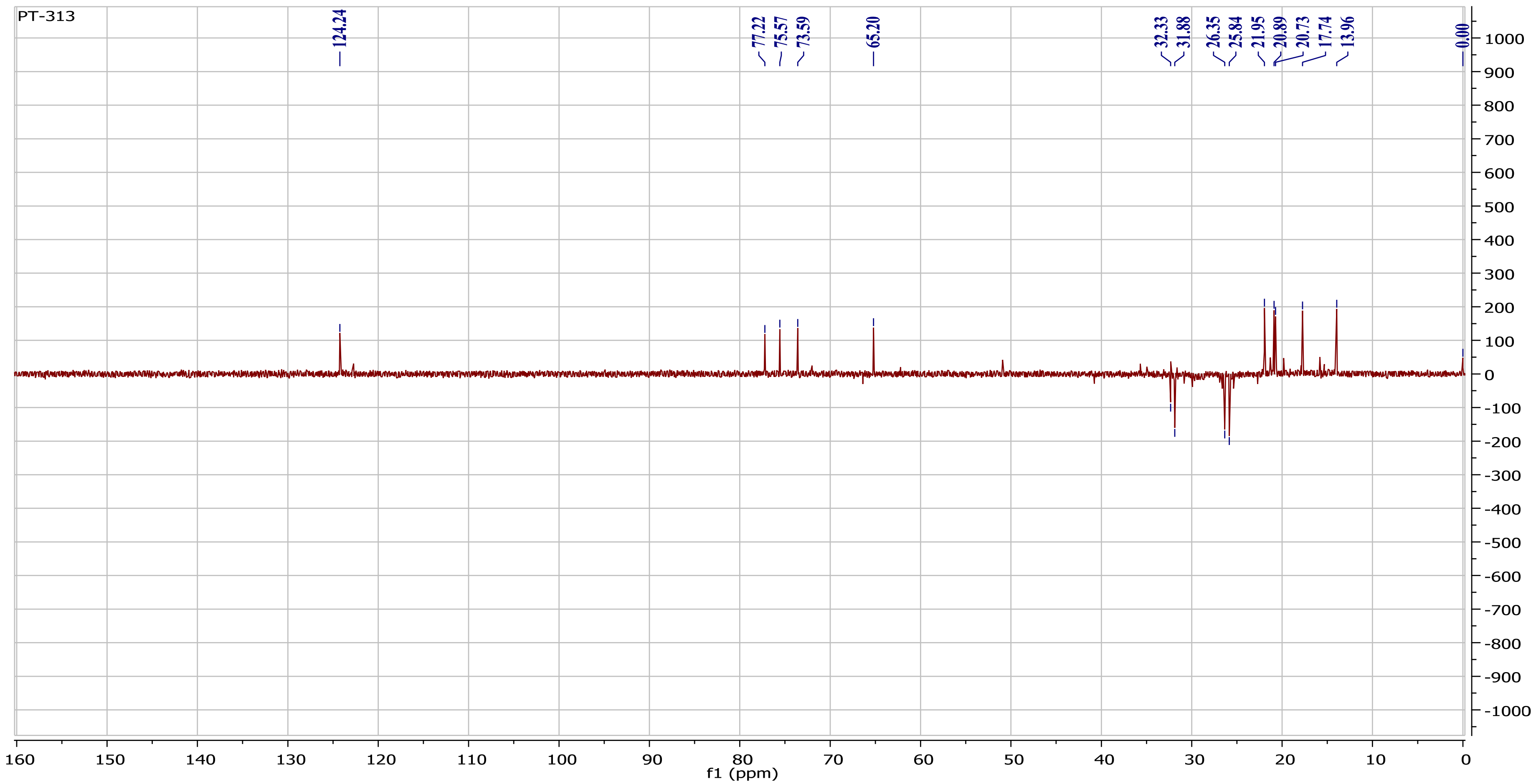

S14: DEPT-135 of **2**

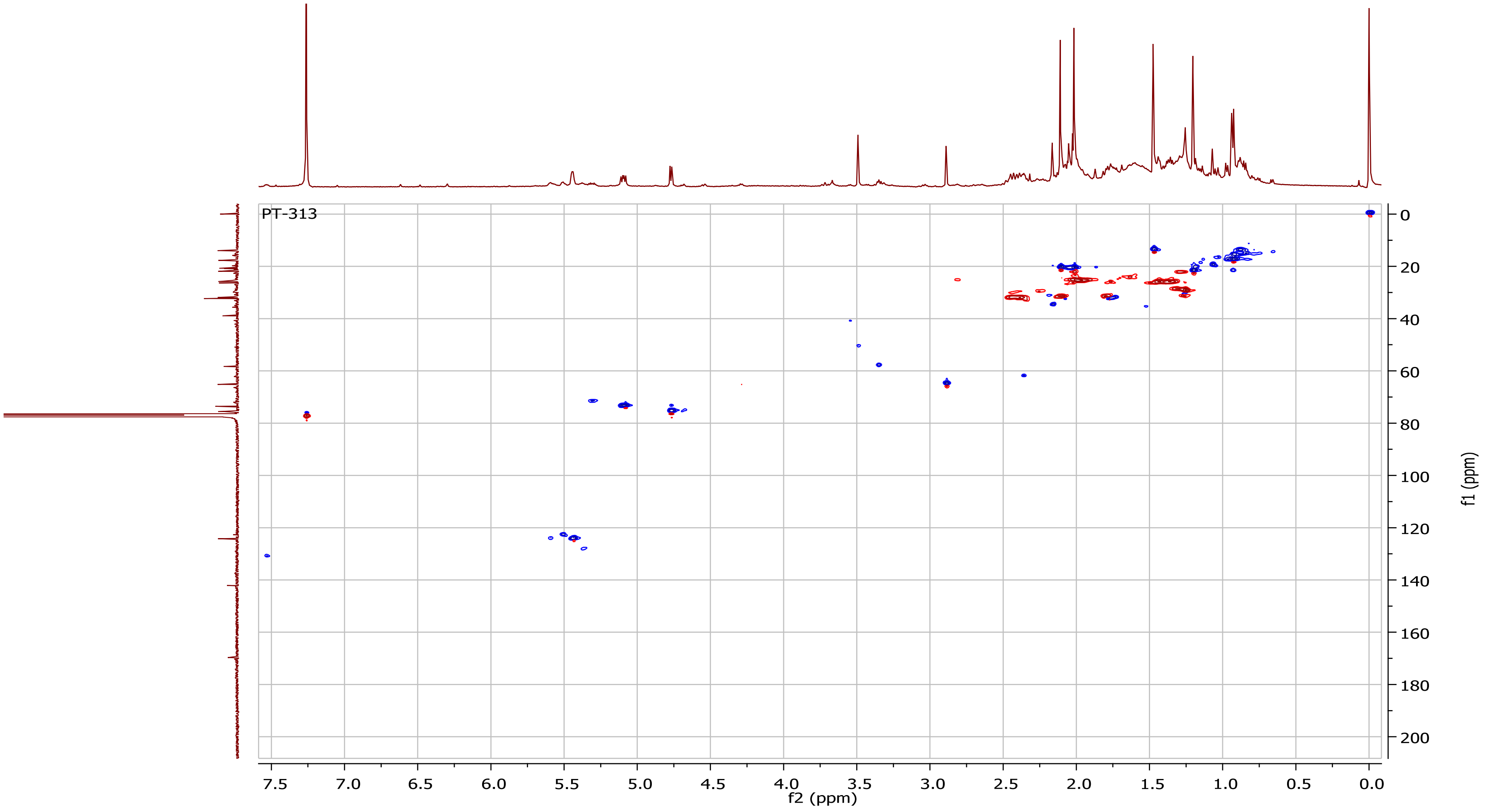

S15: HSQC of **2**

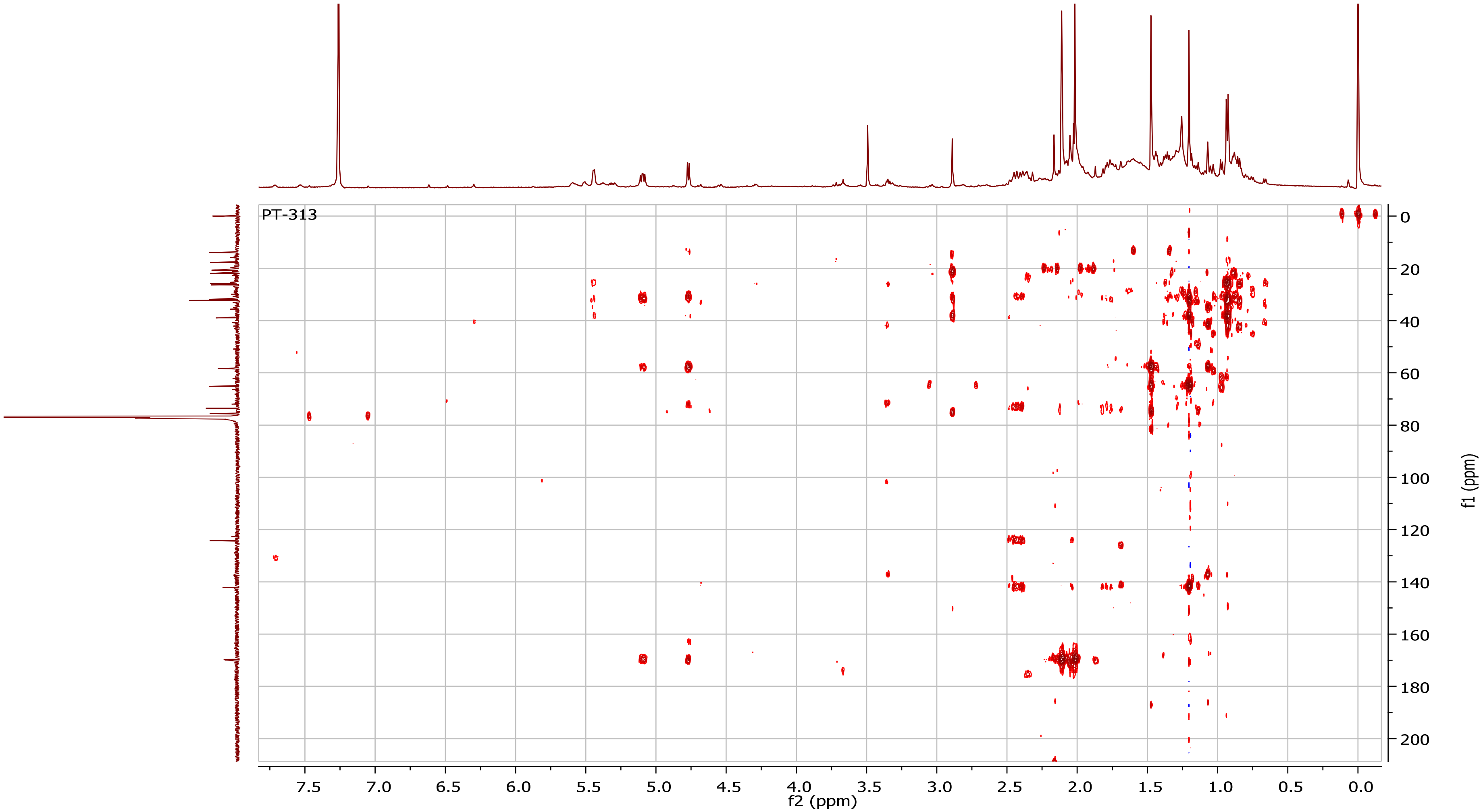
PT-313
f2 (ppm)
f1 (ppm)
7.5
7.0
6.5
6.0
5.5
5.0
4.5
4.0
3.5
3.0
2.5
2.0
1.5
1.0
0.5
0.0
0
20
40
60
80
100
120
140
160
180
200

S16: HMBC of **2**

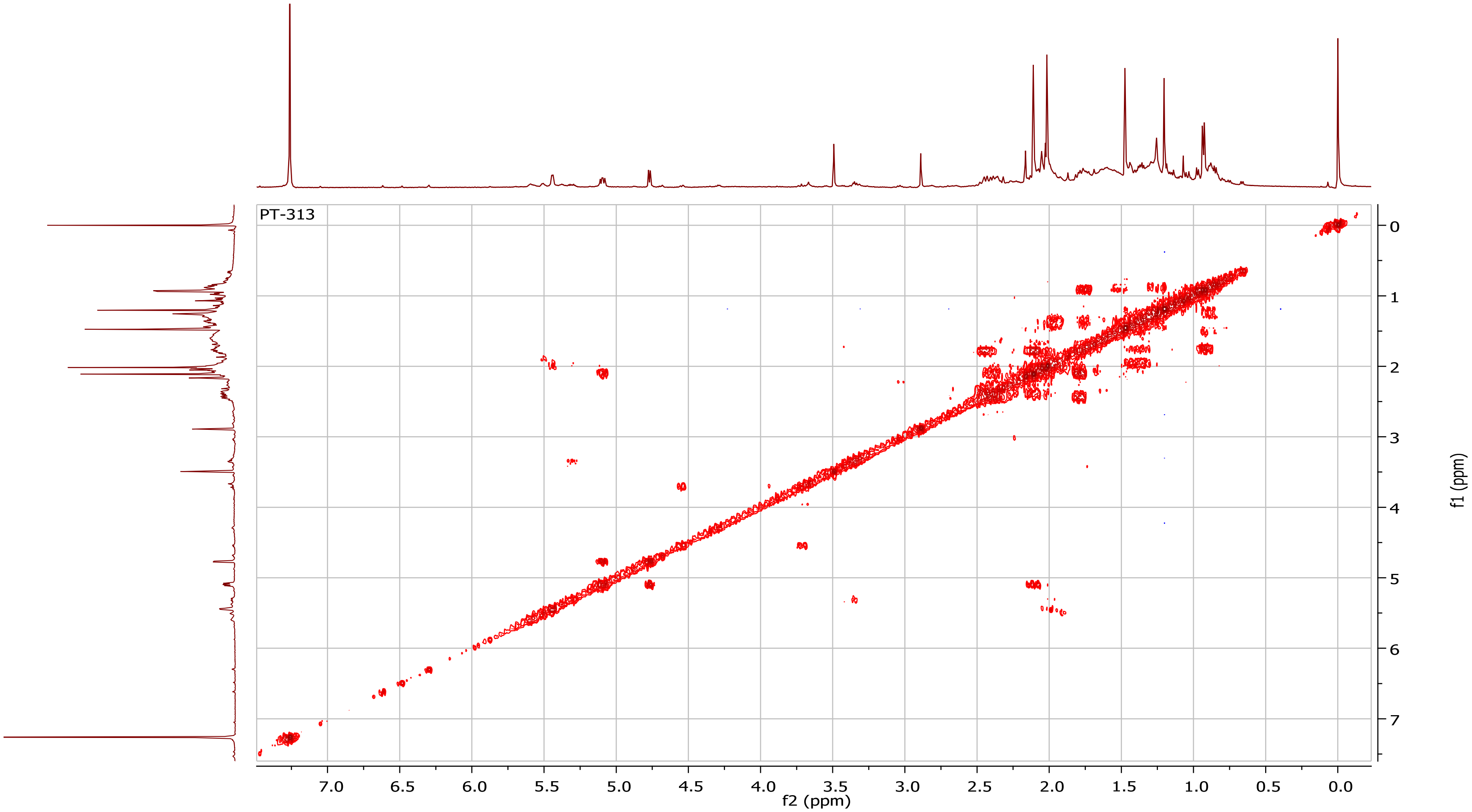

S17: ${}^{1}H$ ${}^{1}H$ COSY of **2**

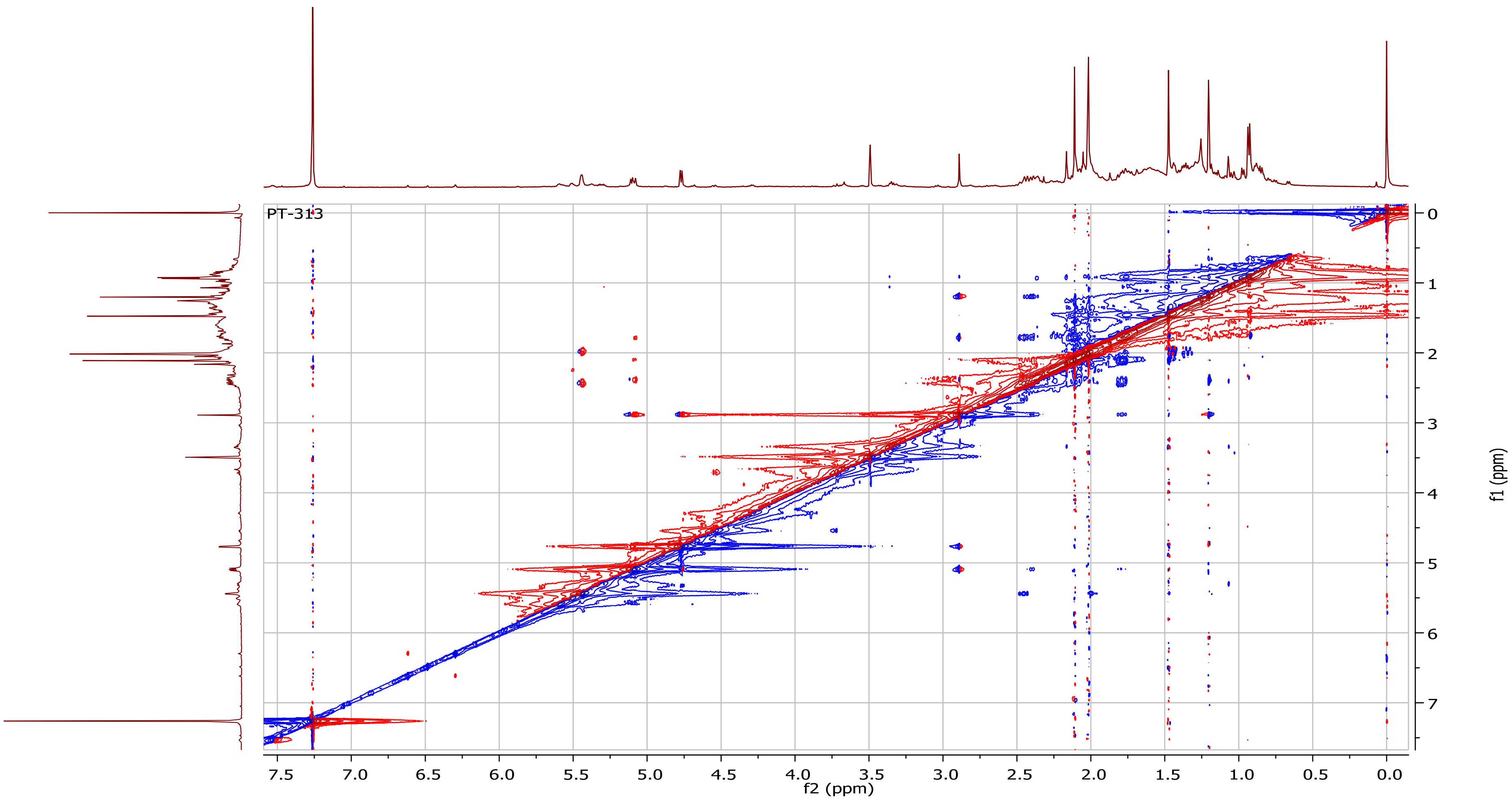

S18: NOESY of **2**

[Mass Spectrum]
Data : Umeyama-CI.17-Aug-2019.003 Date : 17-Aug-2019 10:37
Sample : PT-315(CH4)
Note : MStation
Inlet : Direct Ion Mode : CI+
Spectrum Type : Normal Ion [MF-Linear]
RT : 0.63 min Scan# : 24
BP : m/z 218 Int. : 399.99 (4194240)
Output m/z range : 35 to 500 Cut Level : 0.00 %

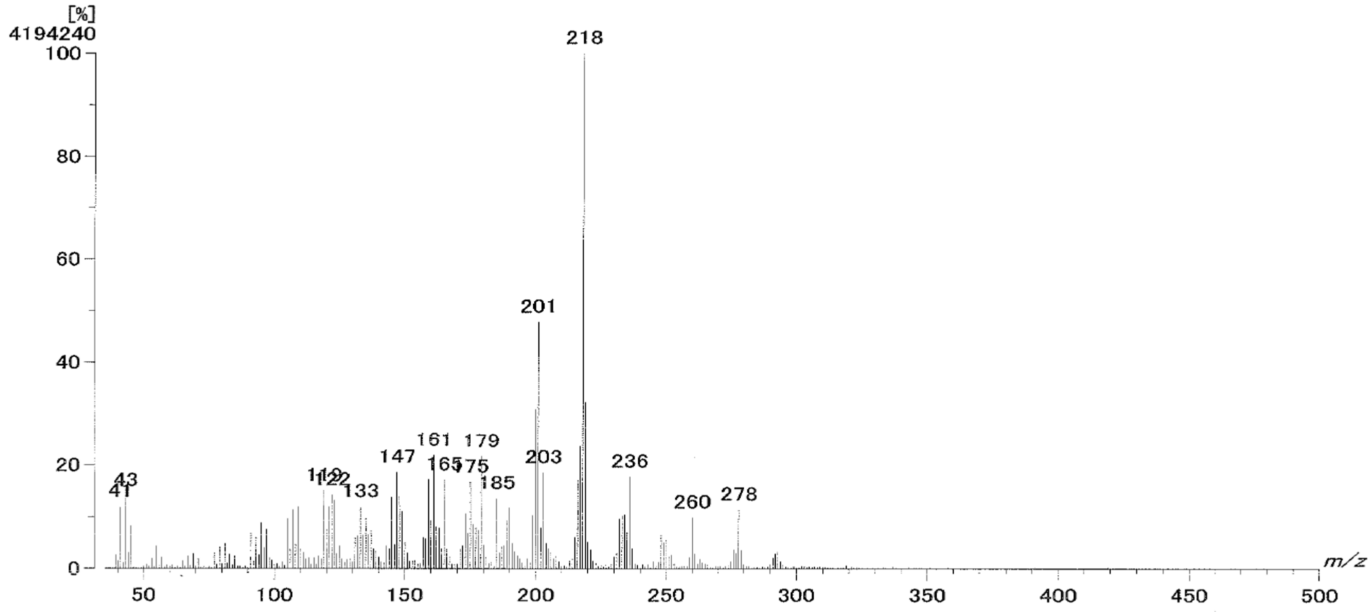

S19: LRCIMS of **3**

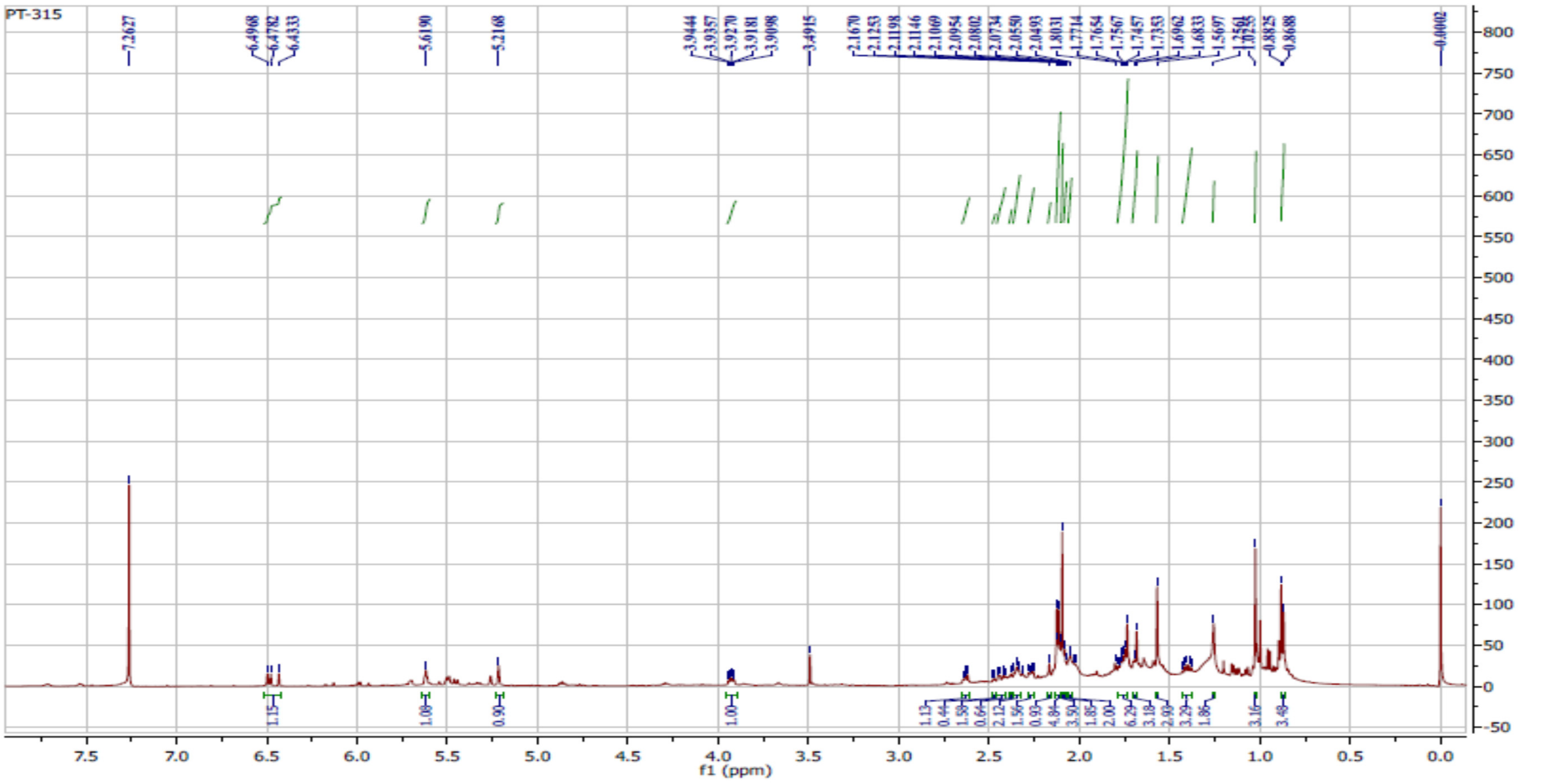

S20: ^{1}H-NMR of **3**

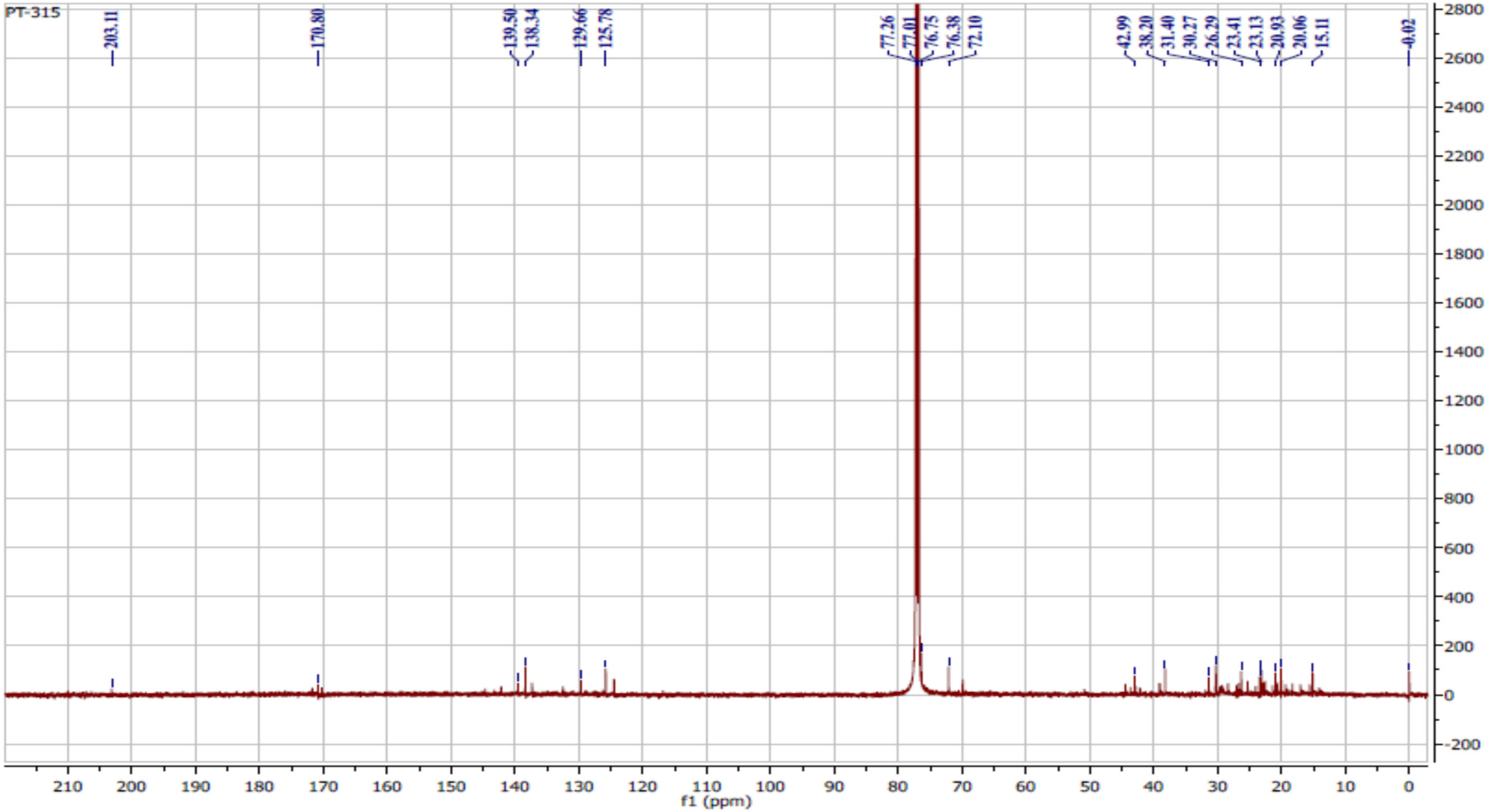

S21: ^{13}C-NMR of **3**

[Mass Spectrum]
Data : Umeyama-CI.29-Jul-2019.002 Date : 29-Jul-2019 08:27
Sample : PT-316(CH4)
Note : MStation
Inlet : Direct Ion Mode : CI+
Spectrum Type : Normal Ion [MF-Linear]
RT : 0.55 min Scan# : 21
BP : m/z 200 Int. : 399.99 (4194240)
Output m/z range : 35 to 500 Cut Level : 0.00 %

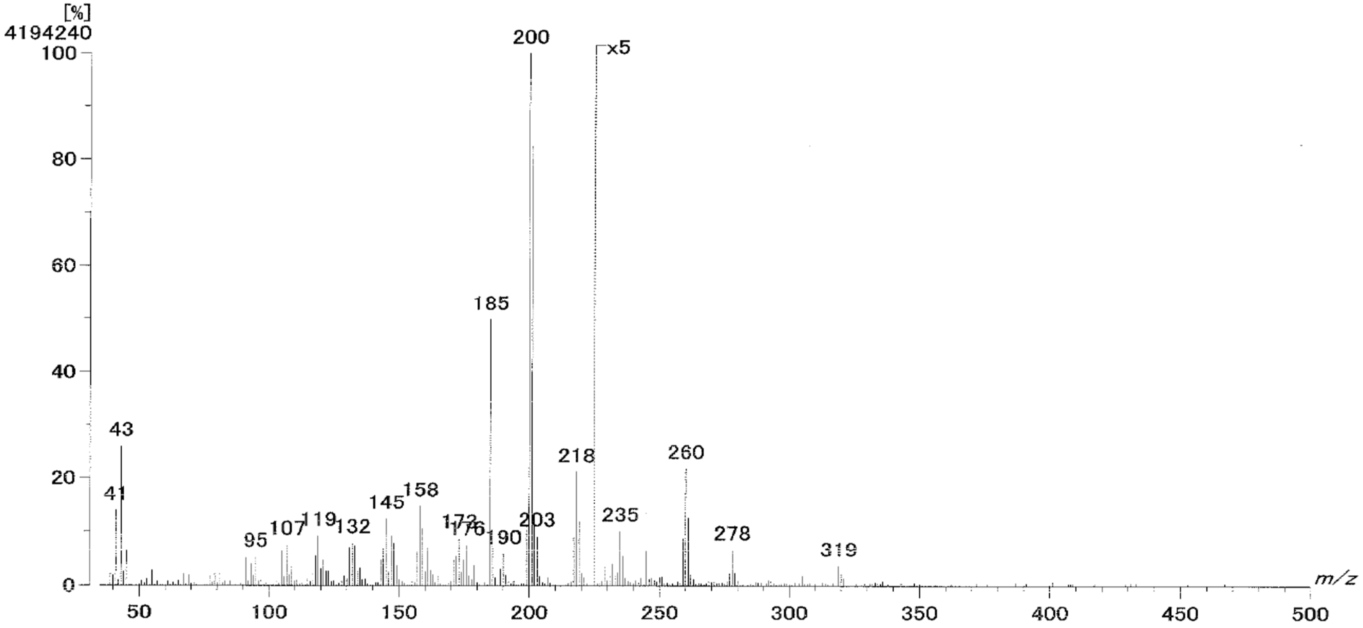

S22: LRCIMS of **4**

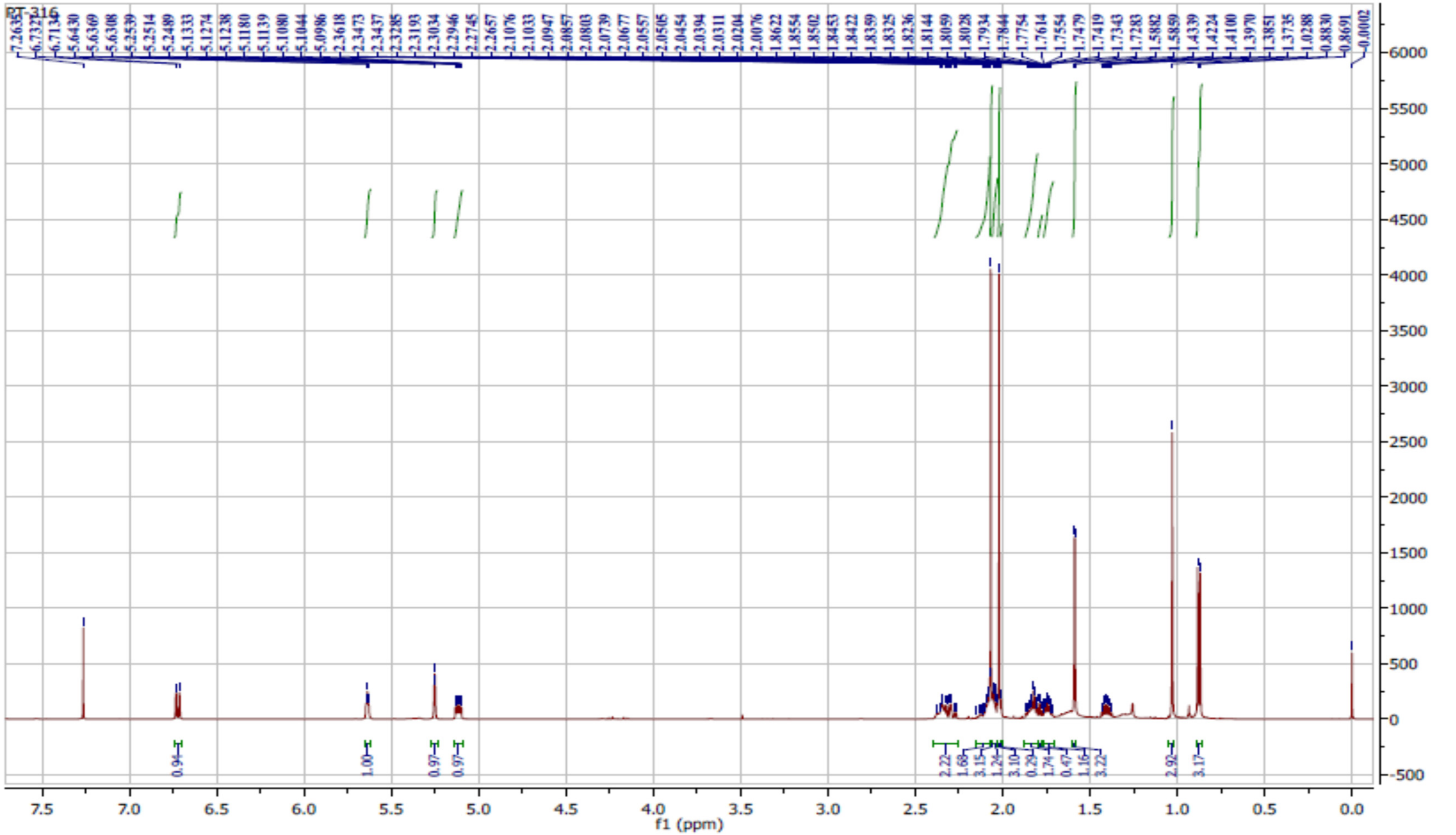

^{1}H-

S23: NMR of **4**

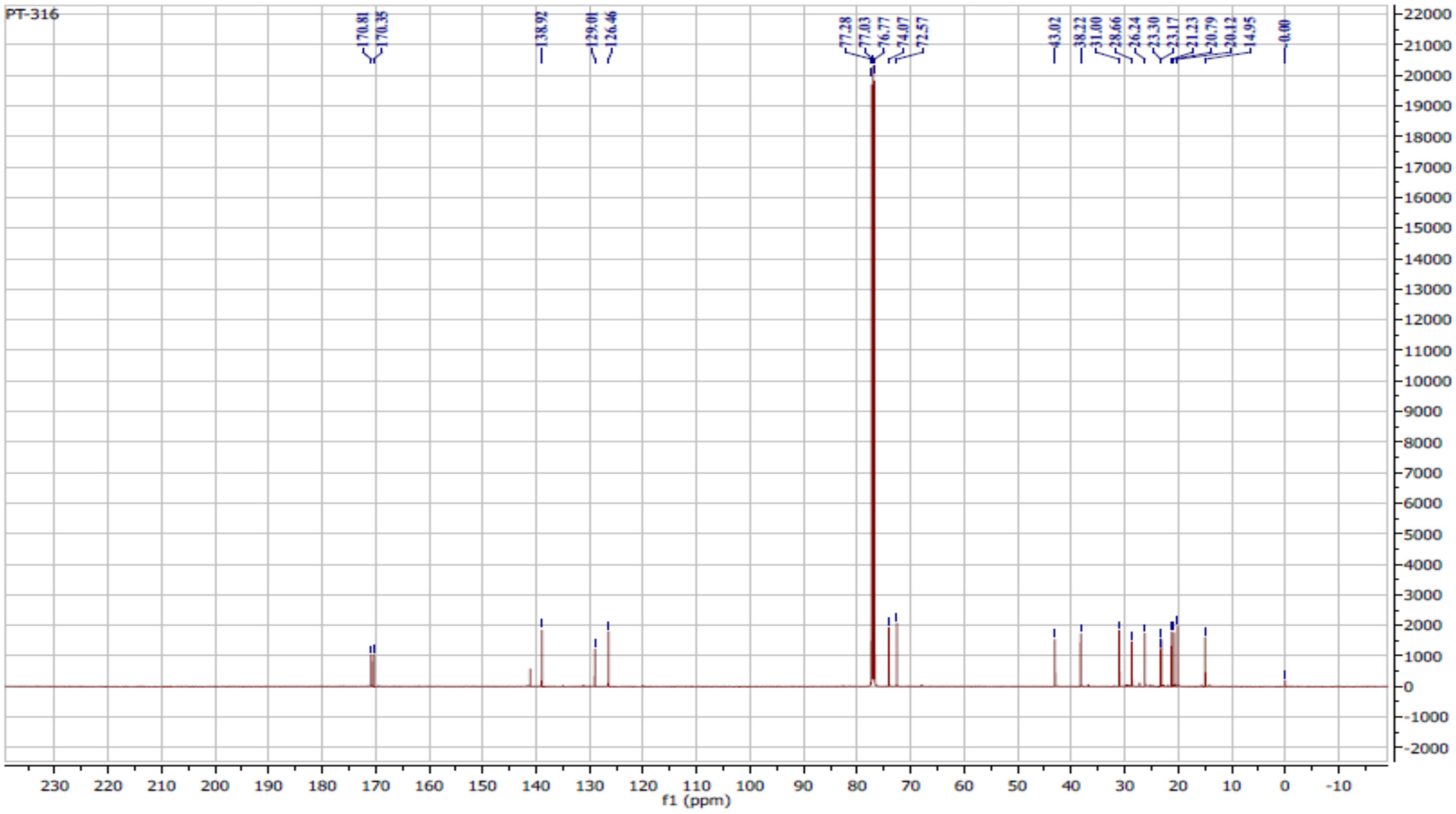

S24: ^{13}C-NMR of **4**

[Mass Spectrum]
Data : Umeyama-EI.22-Jul-2019.001 Date : 22-Jul-2019 09:59
Sample : PT-311
Note : MStation
Inlet : Direct Ion Mode : EI+
Spectrum Type : Normal Ion [MF-Linear]
RT : 0.60 min Scan# : 19
BP : m/z 203 Int. : 1062.04 (11136256)
Output m/z range : 35 to 500 Cut Level : 0.00 %

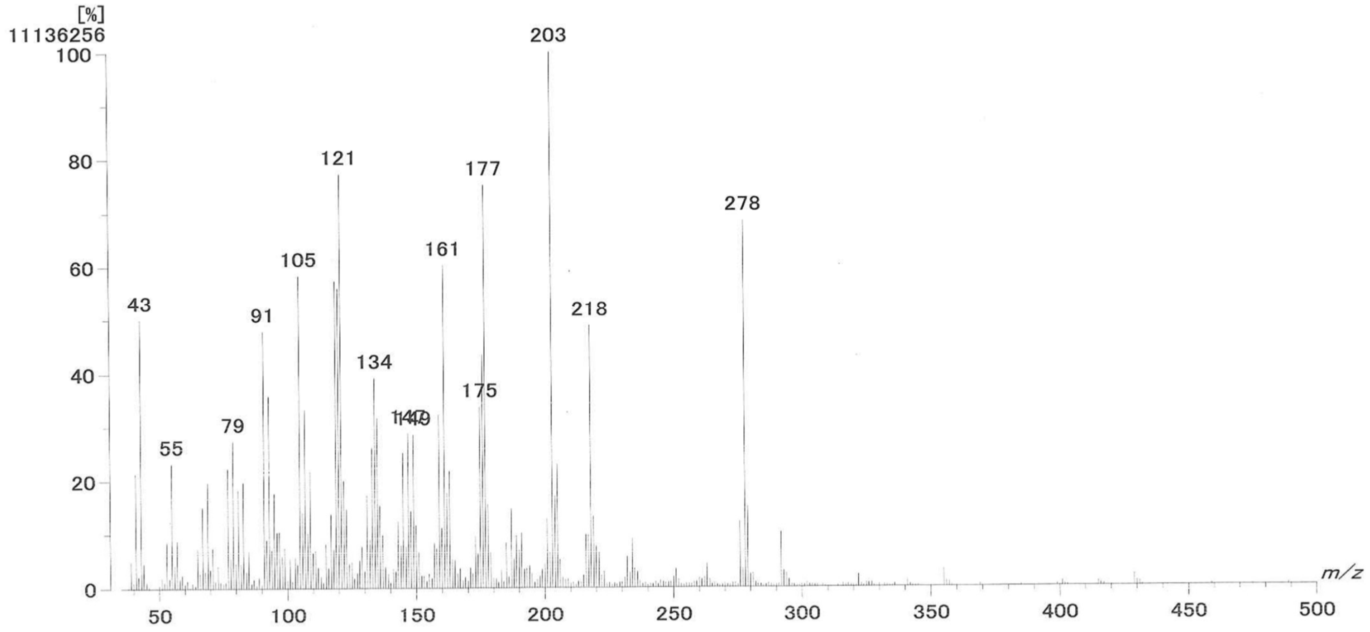

S25: LRCIMS of **5**

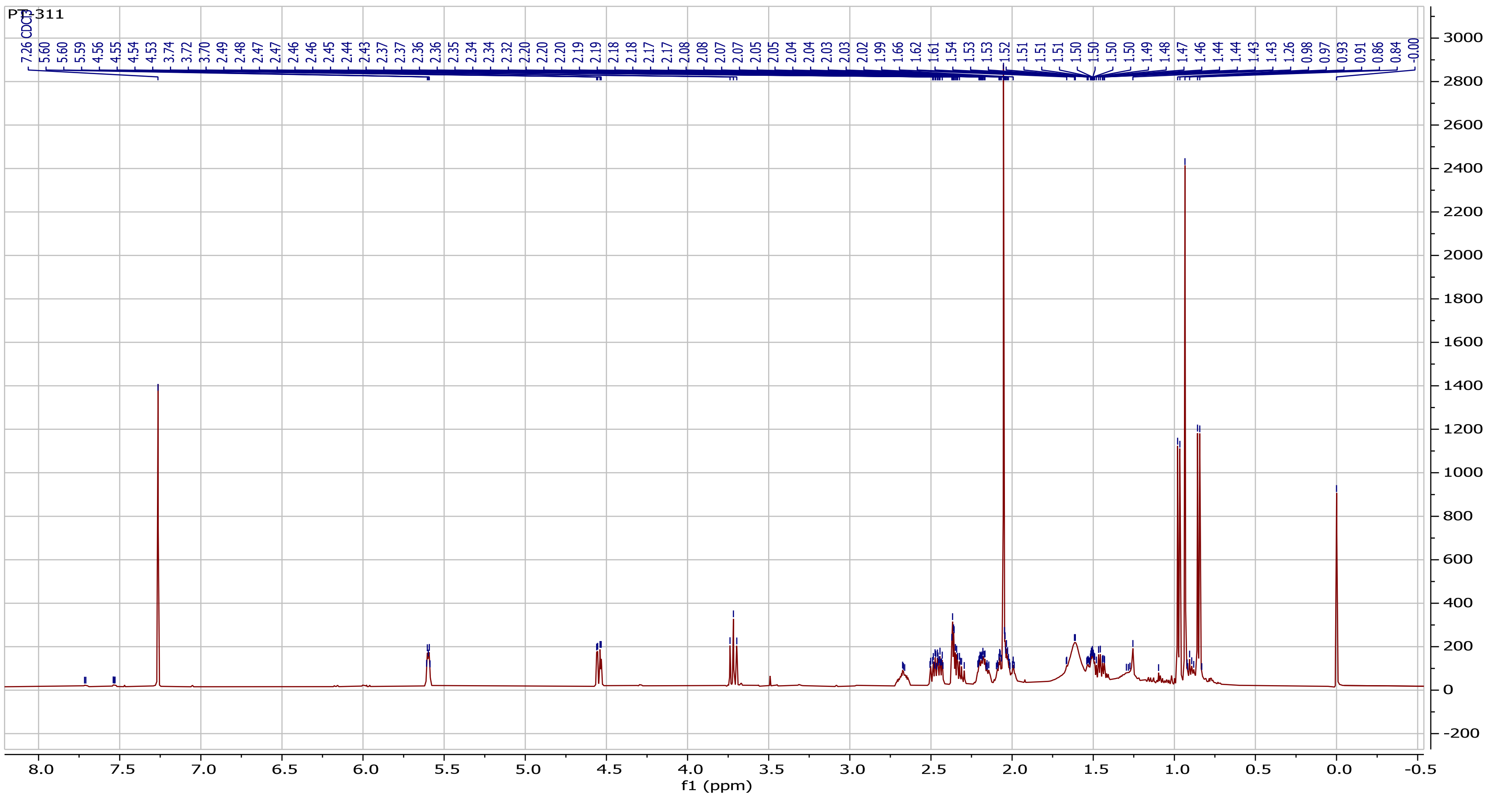

S26: ^{1}H-NMR of **5**

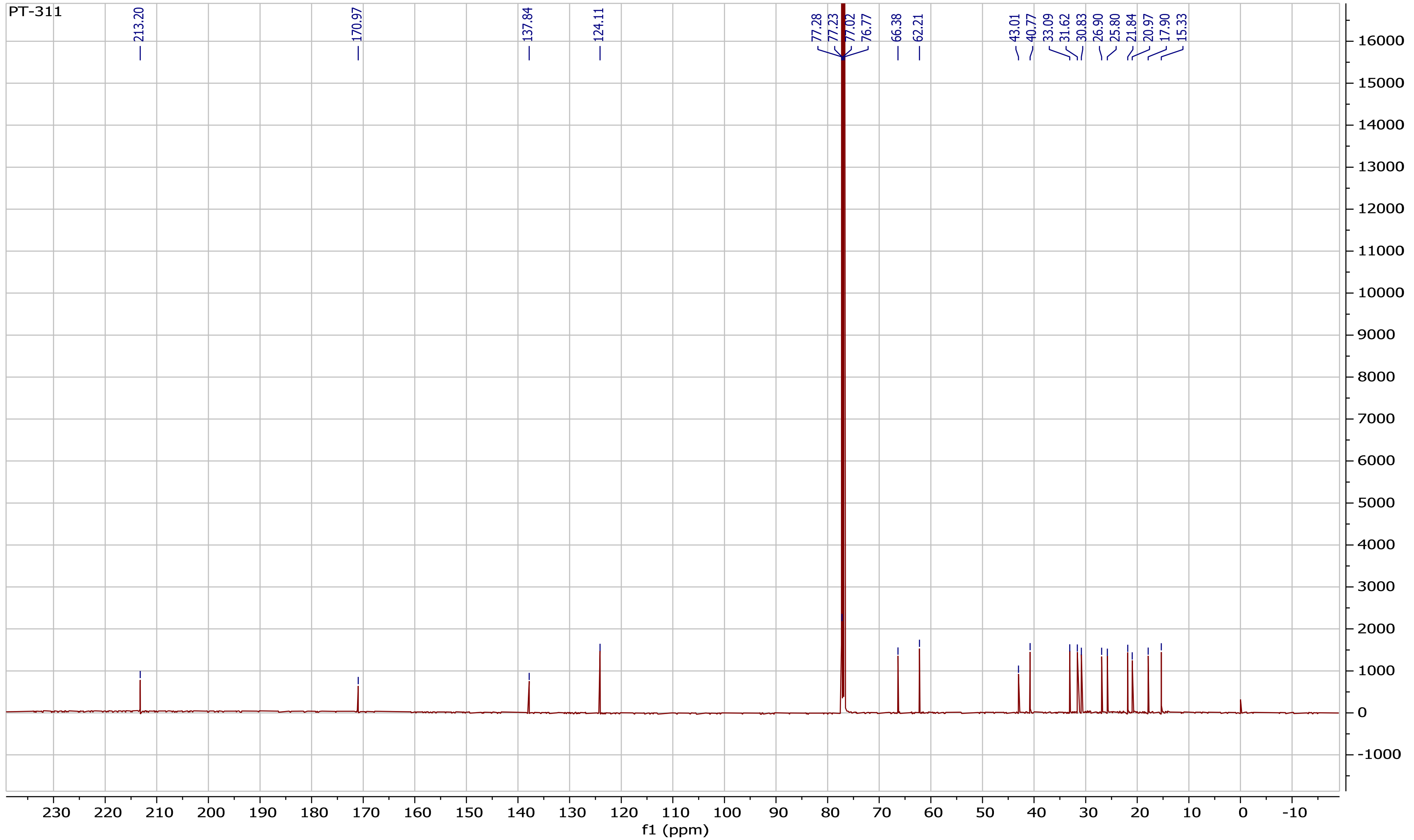

S27: ^{13}C-NMR of **5**

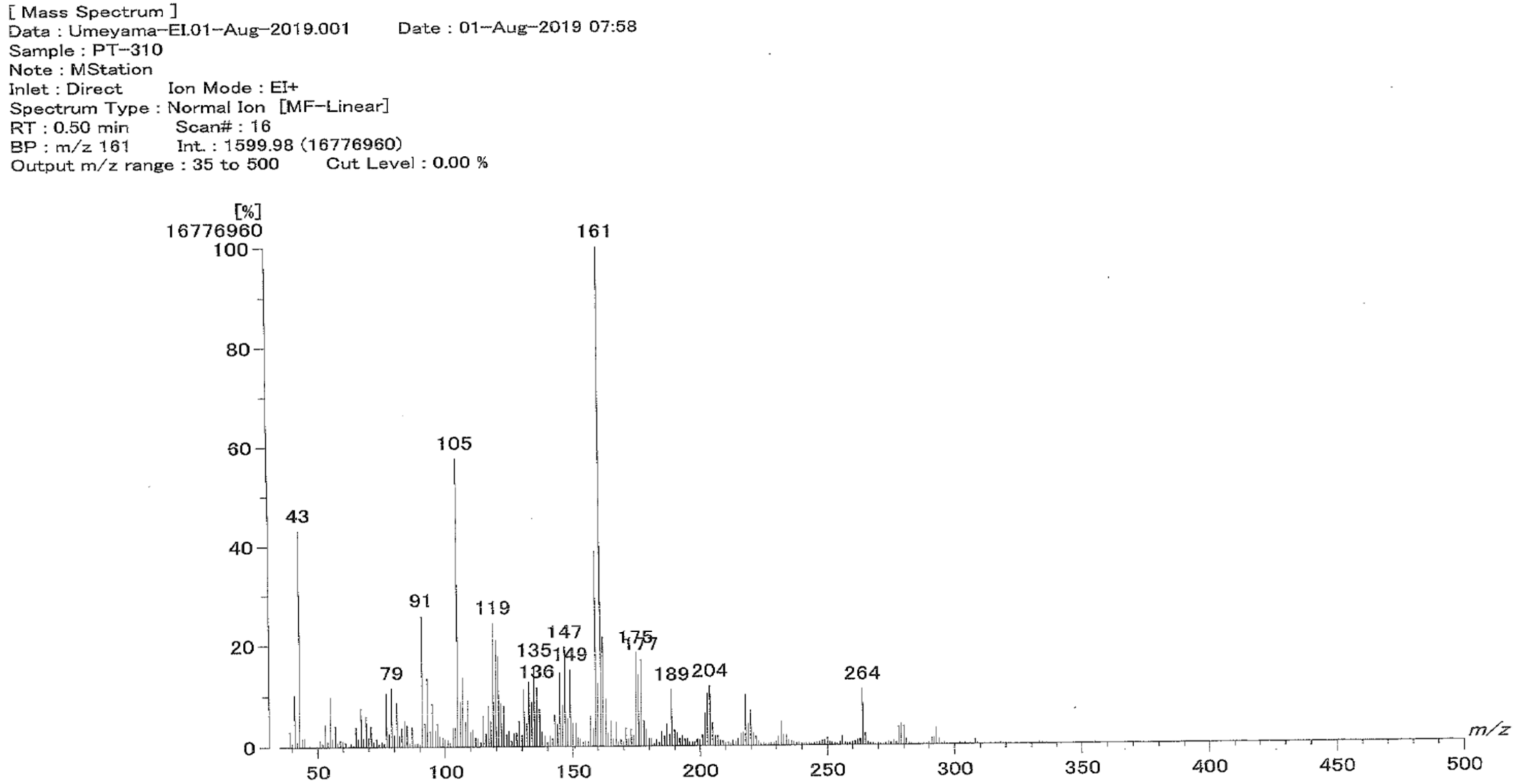

S28: LRCIMS EI-MS of **6**

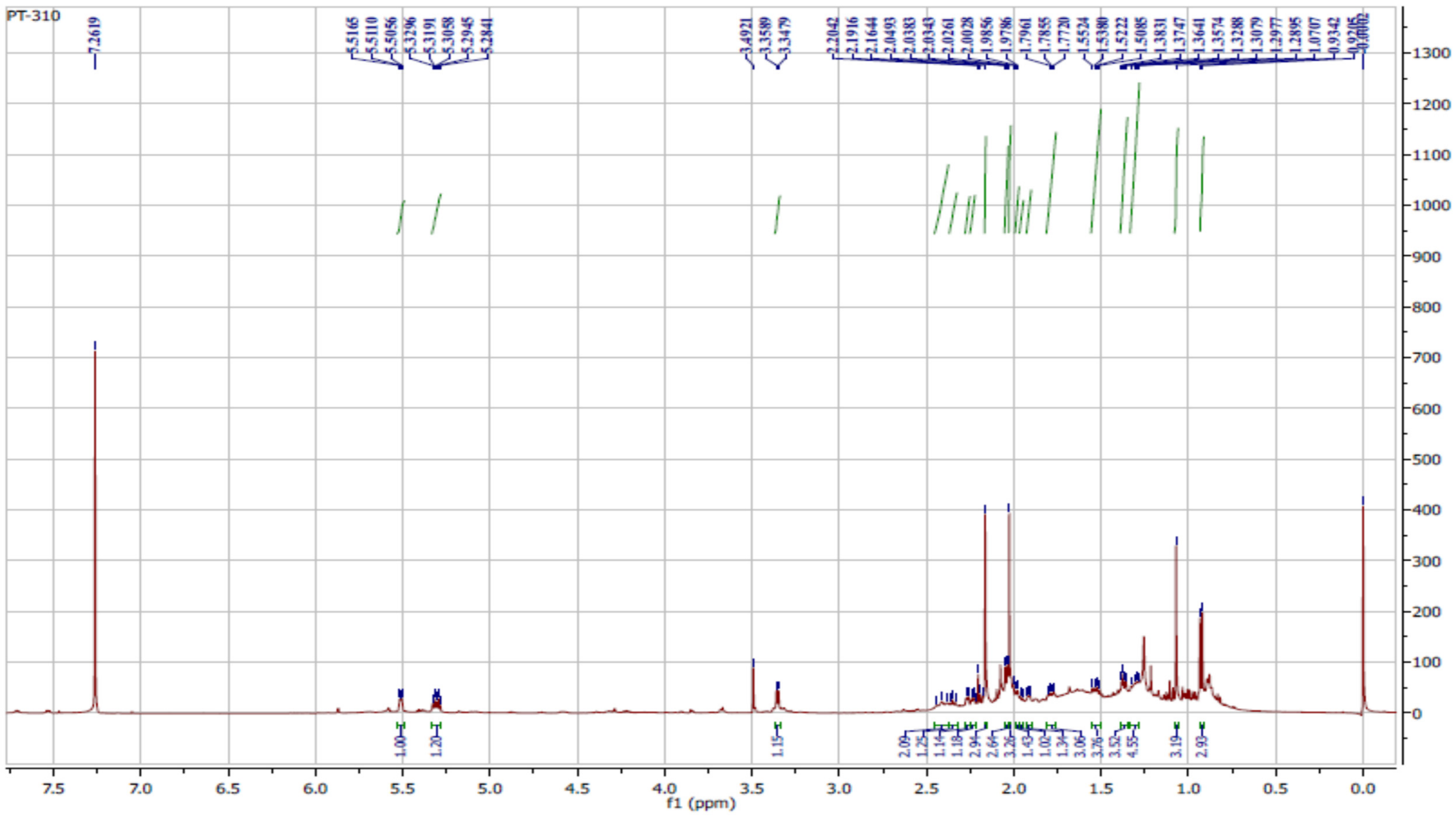

S29: ^{1}H-NMR of **6**

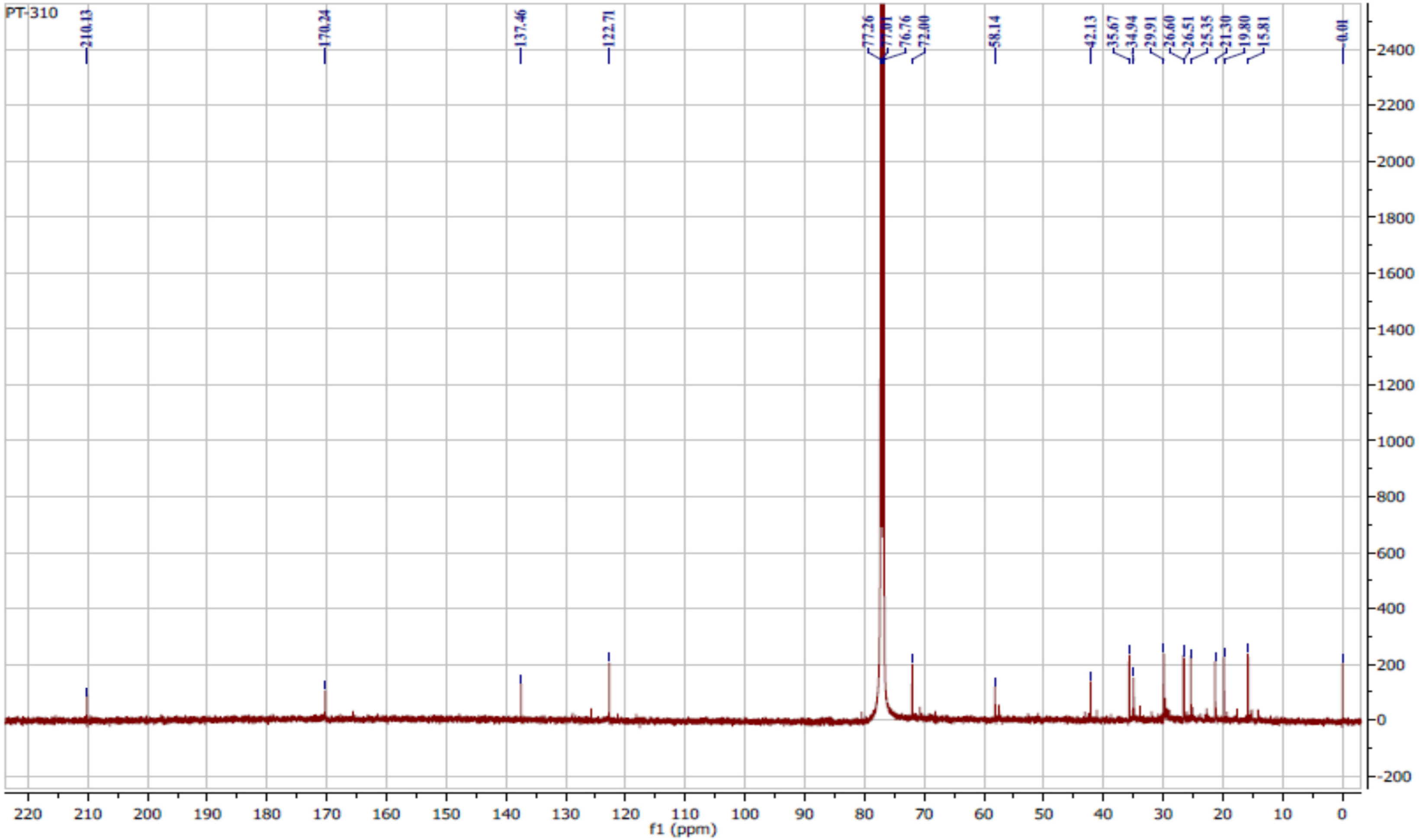

S30: ^{13}C-NMR of **6**

[Mass Spectrum]
Data : Umeyama-CI.17-Aug-2019.004   Date : 17-Aug-2019 10:44
Sample : PT-39(CH4)
Note : MStation
Inlet : Direct   Ion Mode : CI+
Spectrum Type : Normal Ion [MF-Linear]
RT : 0.79 min   Scan# : 30
BP : m/z 233   Int. : 114.96 (1205440)
Output m/z range : 35 to 500   Cut Level : 0.00 %

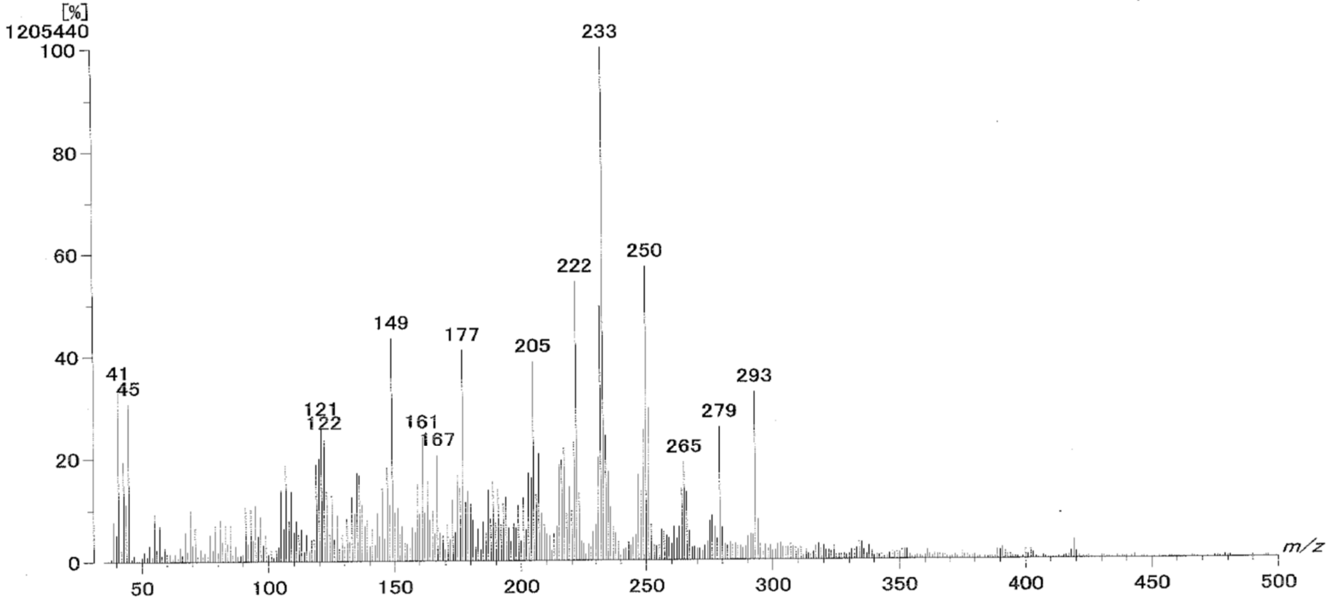

S31: LRCIMS EI-MS of **7**

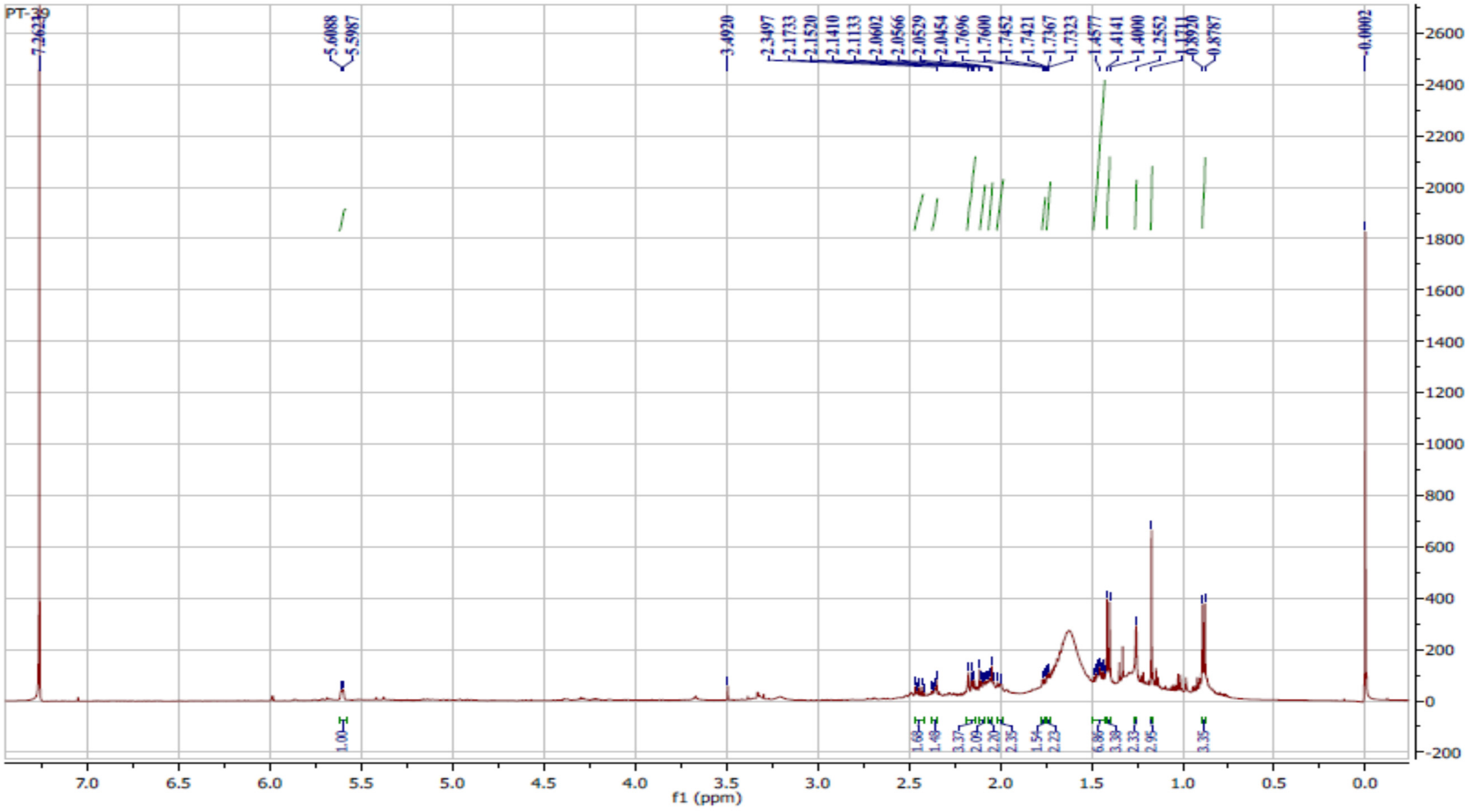

S32: ^{1}H-NMR of **7**

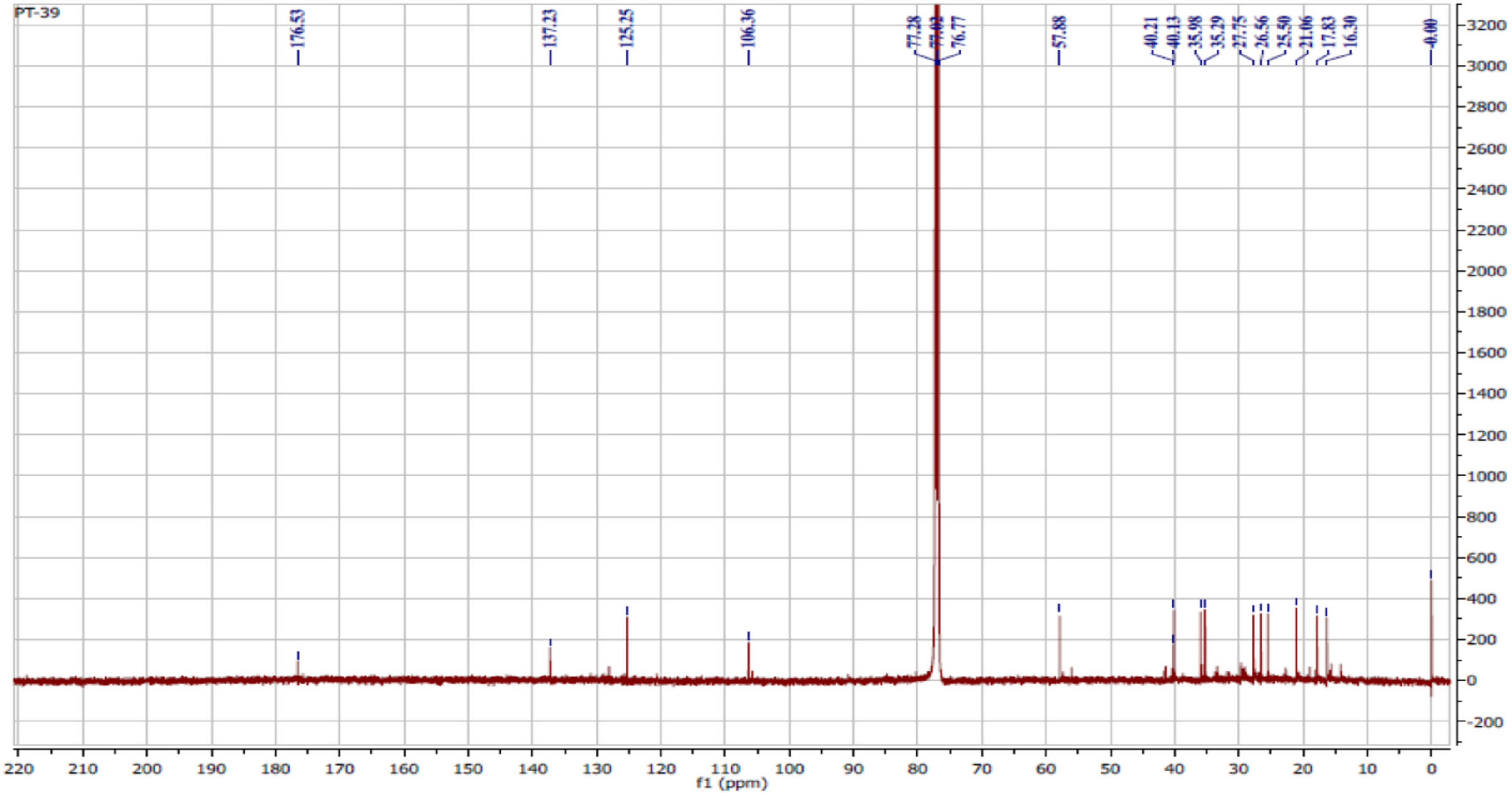

S33:^{13}C-NMR of **7**

[Mass Spectrum]
Data : Umeyama-EI.17-Jul-2019.004 Date : 17-Jul-2019 15:21
Sample : PT-42
Note : MStation
Inlet : Direct Ion Mode : EI+
Spectrum Type : Normal Ion [MF-Linear]
RT : 0.50 min Scan# : 16
BP : m/z 43 Int. : 1466.07 (15372800)
Output m/z range : 35 to 500 Cut Level : 0.00 %

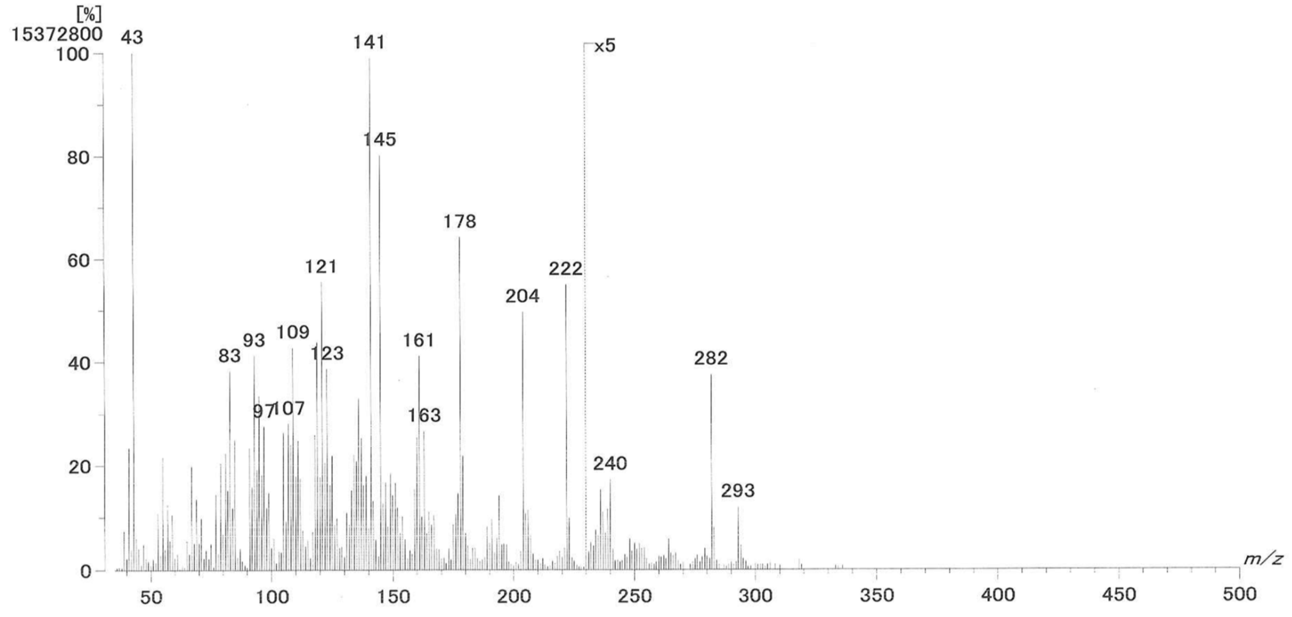

S34:LRCIMS EI-MS of **8**

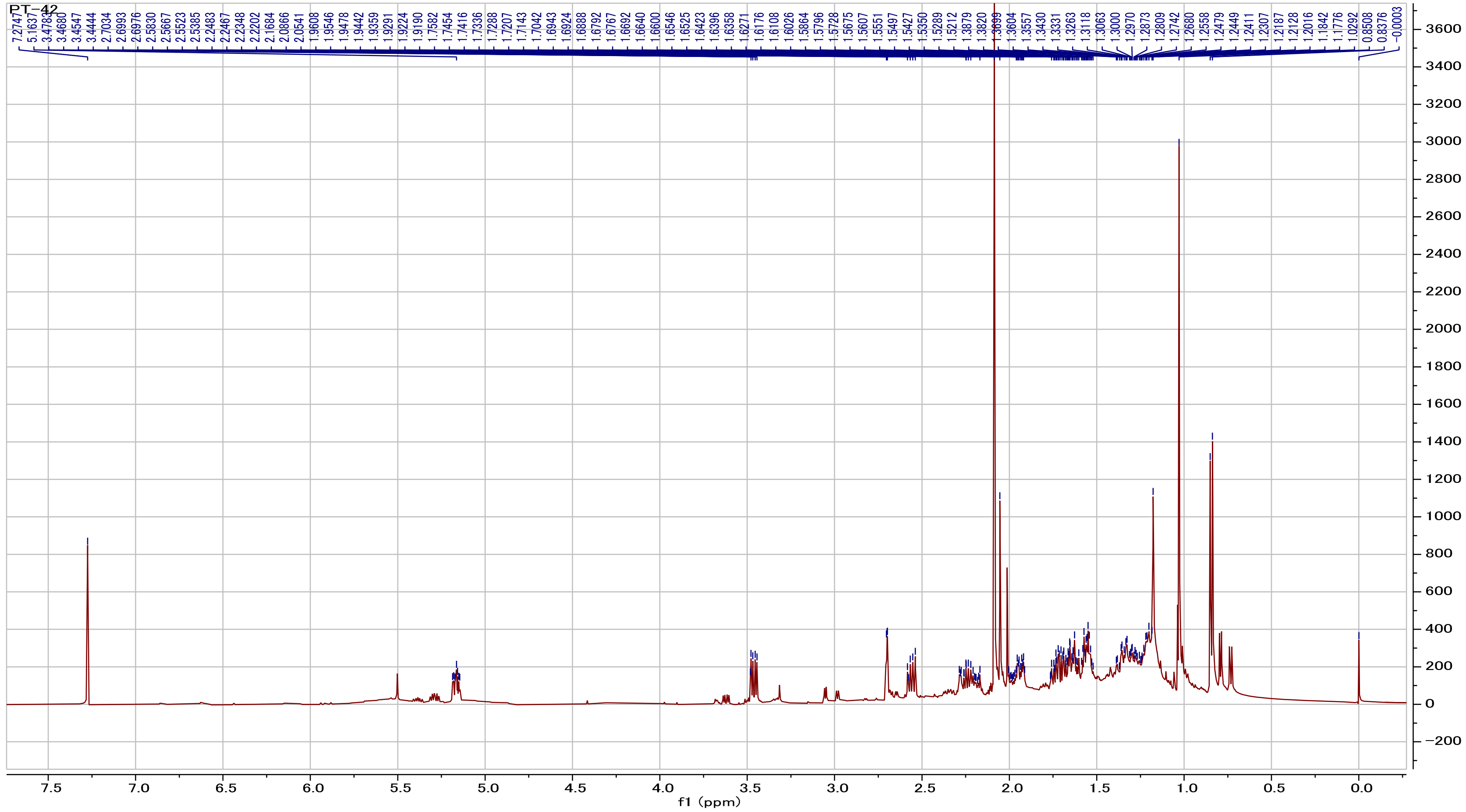

S35: ^{1}H-NMR of **8**

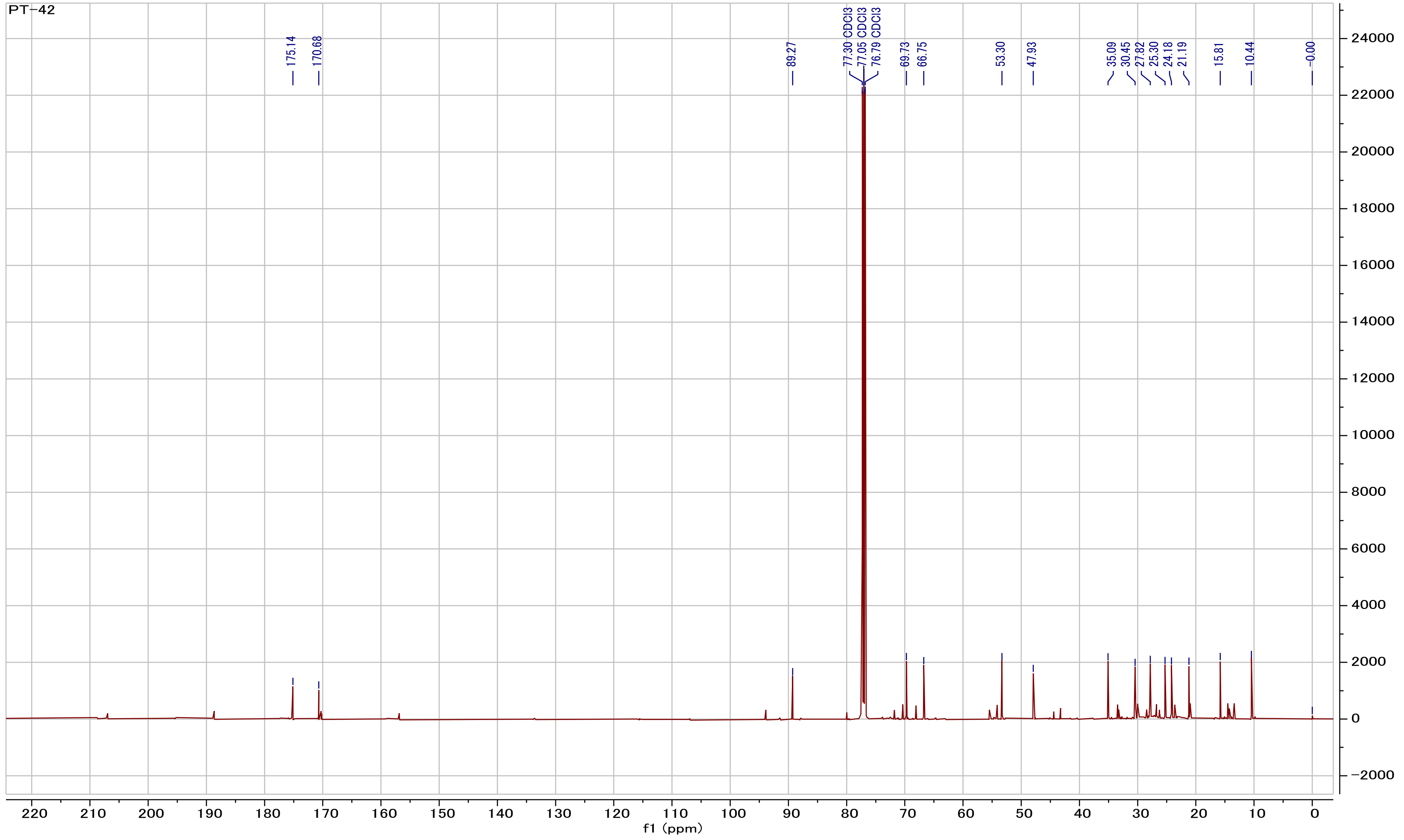

S36: ^{13}C-NMR of **8**

[Mass Spectrum]
Data : Umeyama-EI.17-Jul-2019.005 Date : 17-Jul-2019 15:27
Sample : PT-45
Note : MStation
Inlet : Direct Ion Mode : EI+
Spectrum Type : Normal Ion [MF-Linear]
RT : 0.47 min Scan# : 15
BP : m/z 177 Int. : 1599.98 (16776960)
Output m/z range : 35 to 500 Cut Level : 0.00 %

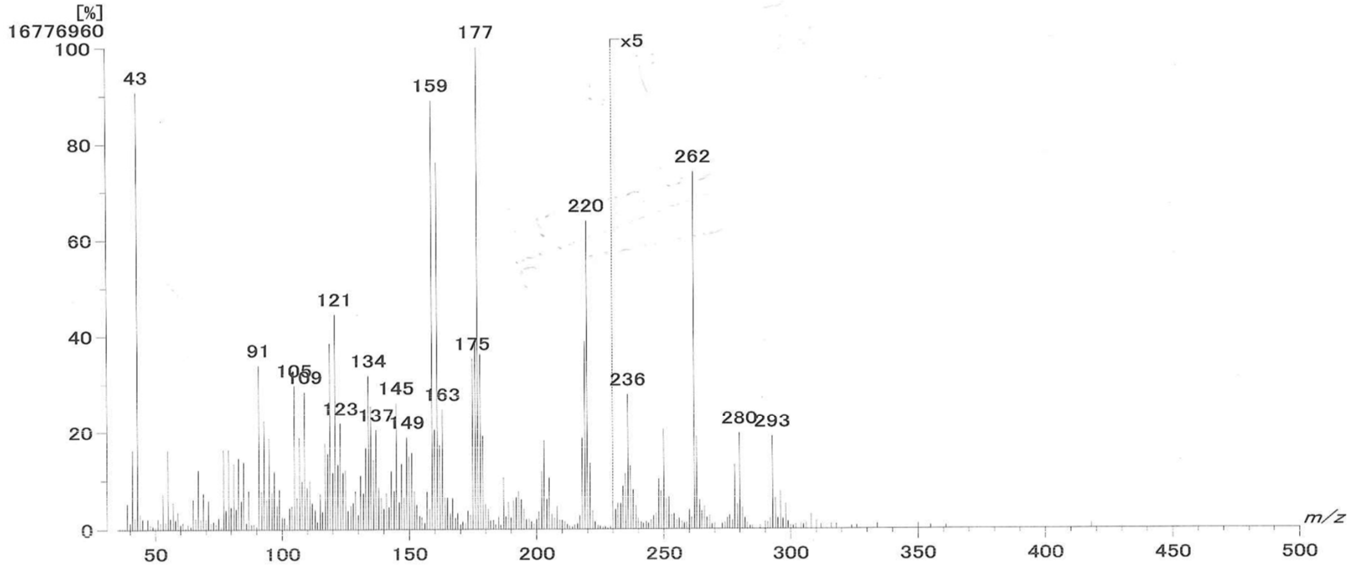

S37: LRCIMS EI-MS of **9**

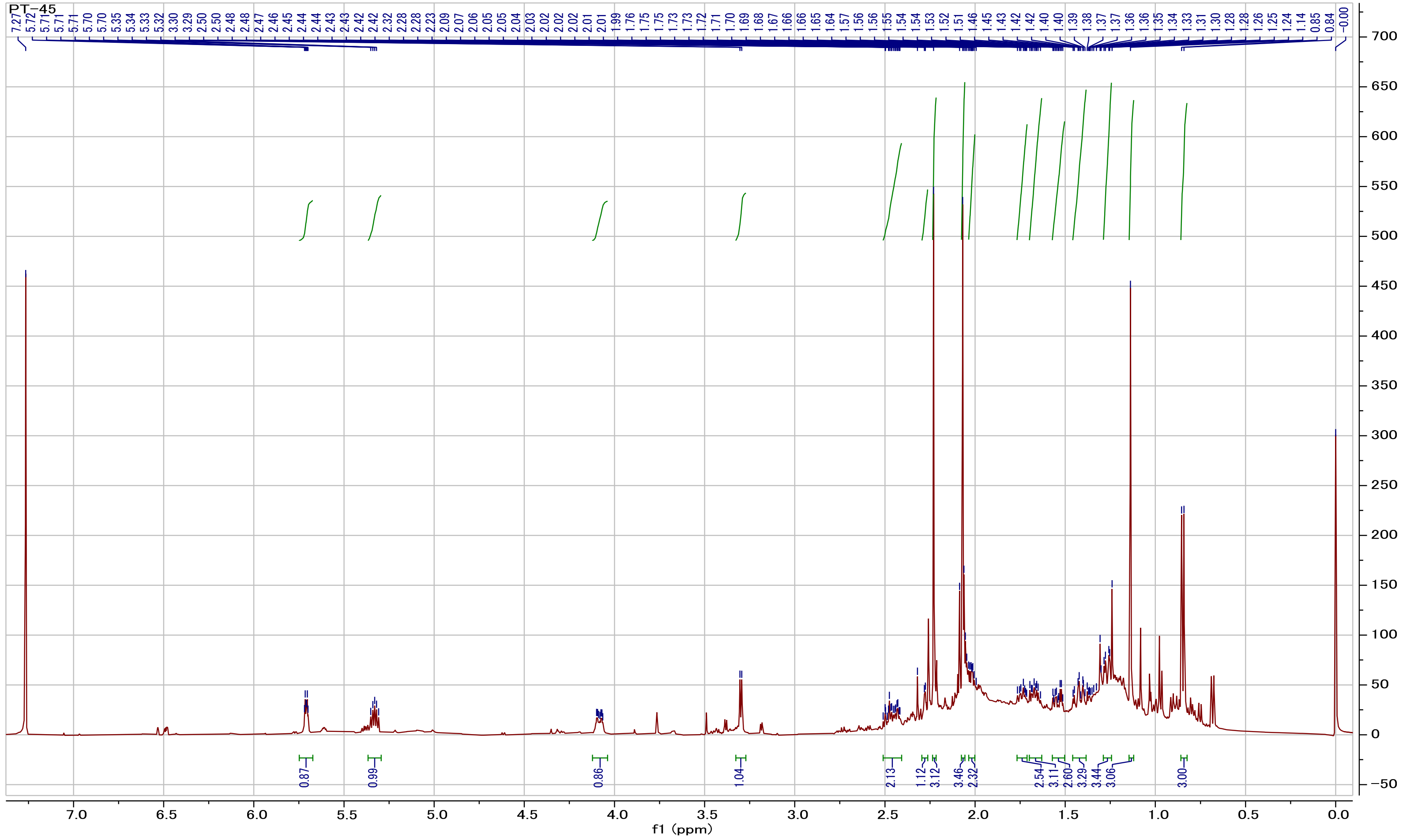

S38: ^{1}H-NMR of **9**

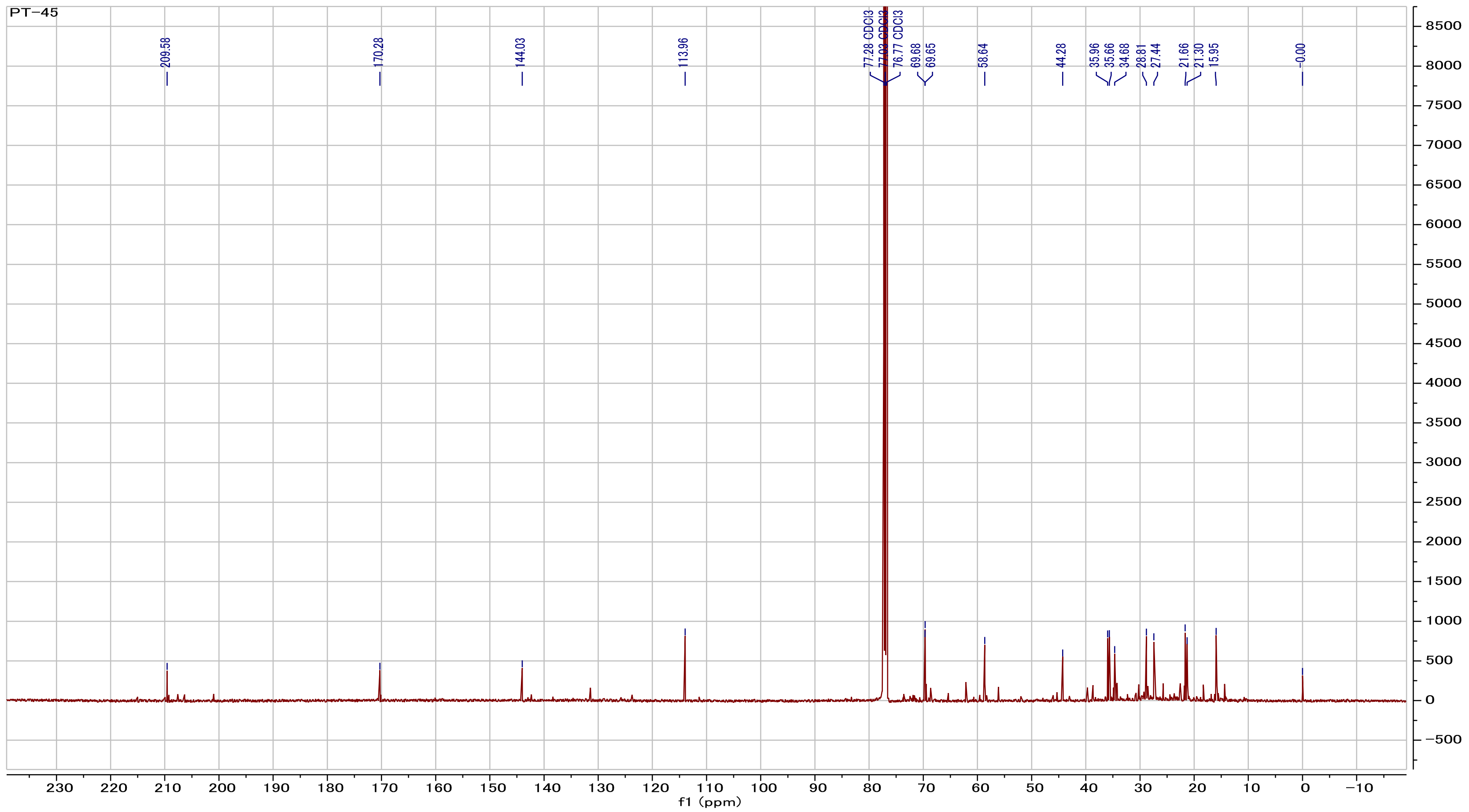

S39: ^{13}C-NMR of **9**

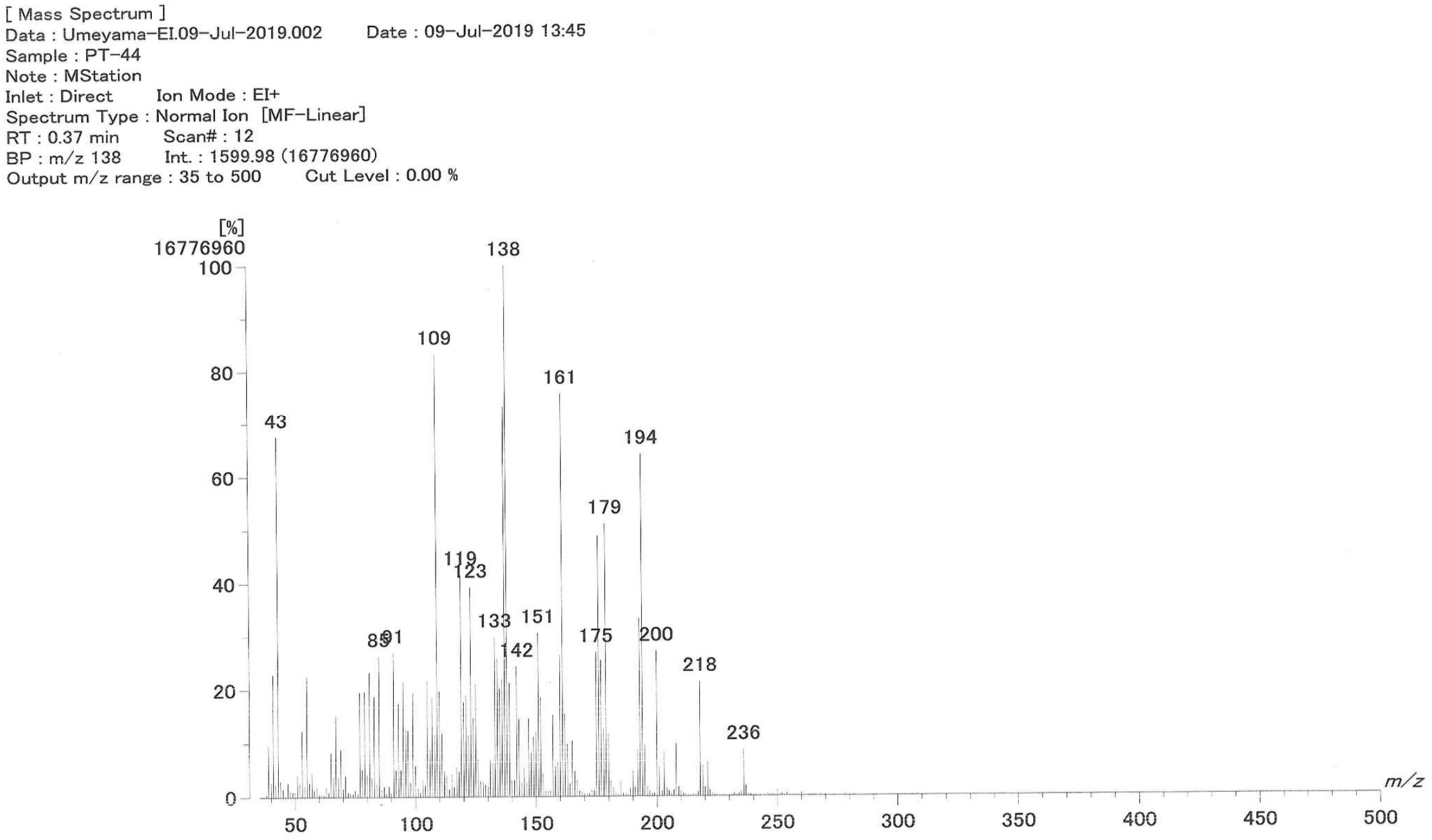

S40: LRCIMS EI-MS of **10**

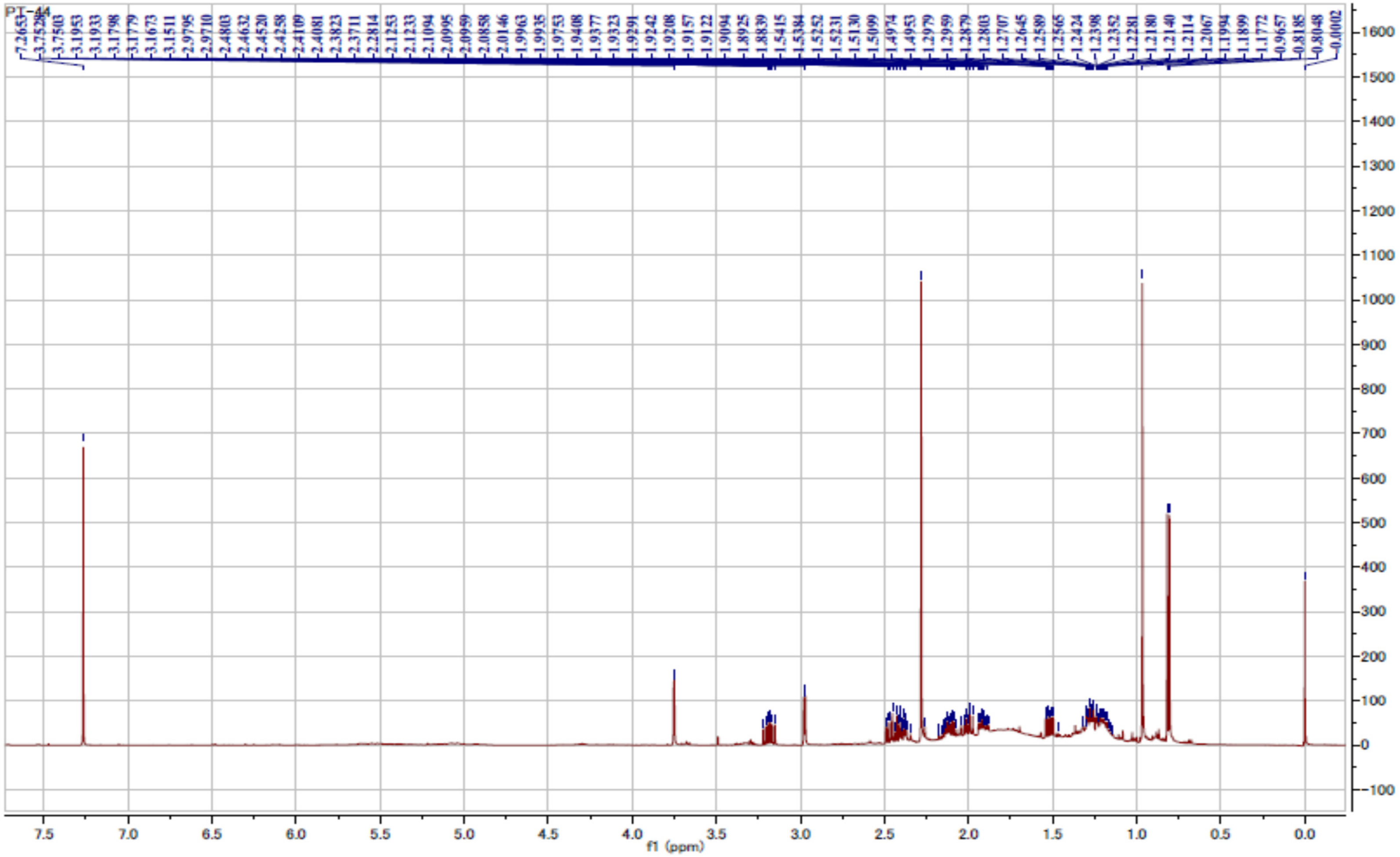

S41: ^{1}H-NMR of **10**

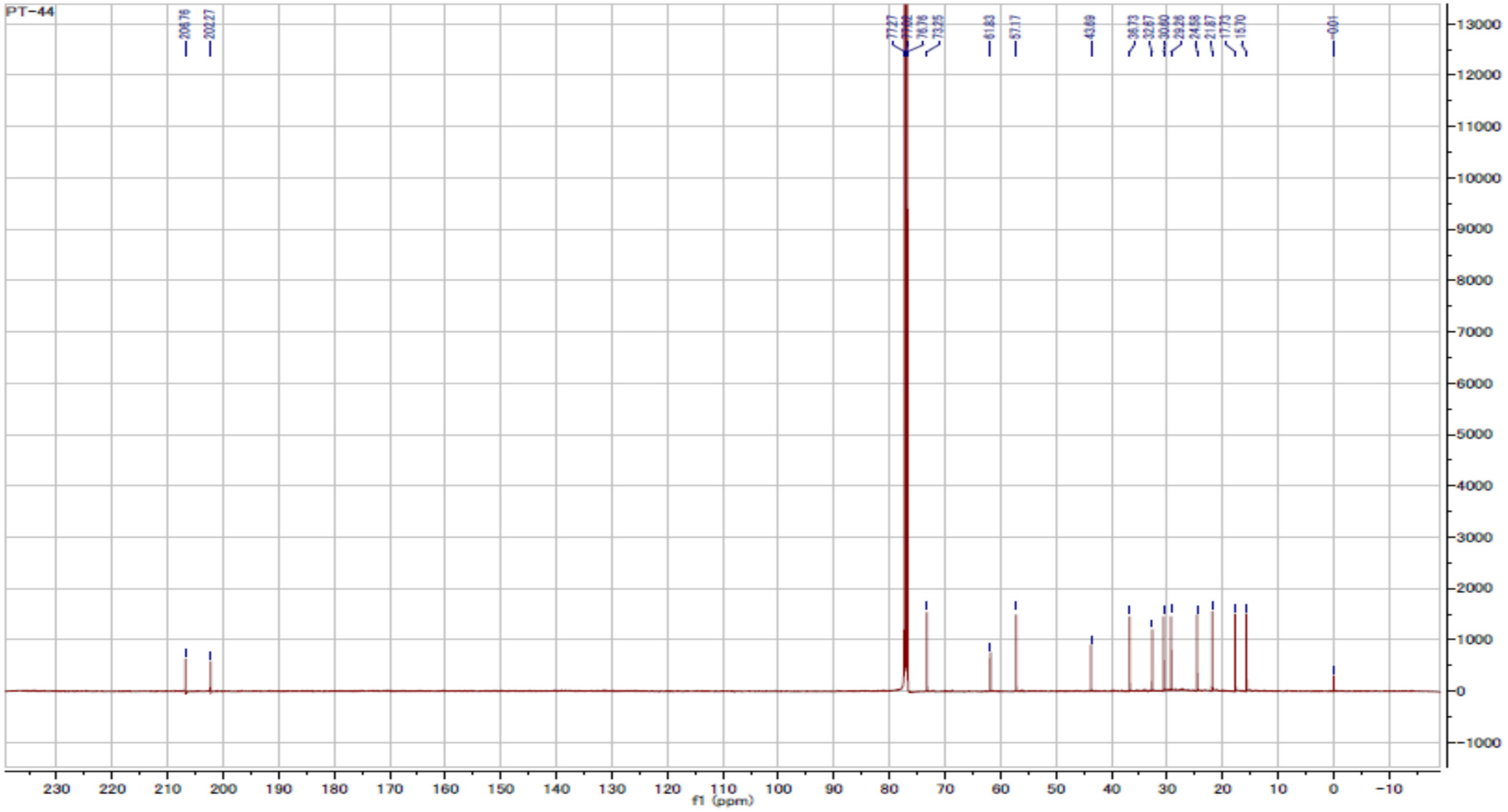

S42: ^{13}C-NMR of **10**

[Mass Spectrum]
Data : Umeyama-EI.24-Jul-2019.001 Date : 24-Jul-2019 16:28
Sample : PT-34
Note : MStation
Inlet : Direct Ion Mode : EI+
Spectrum Type : Normal Ion [MF-Linear]
RT : 0.57 min Scan# : 18
BP : m/z 43 Int. : 1599.98 (16776960)
Output m/z range : 35 to 500 Cut Level : 0.00 %

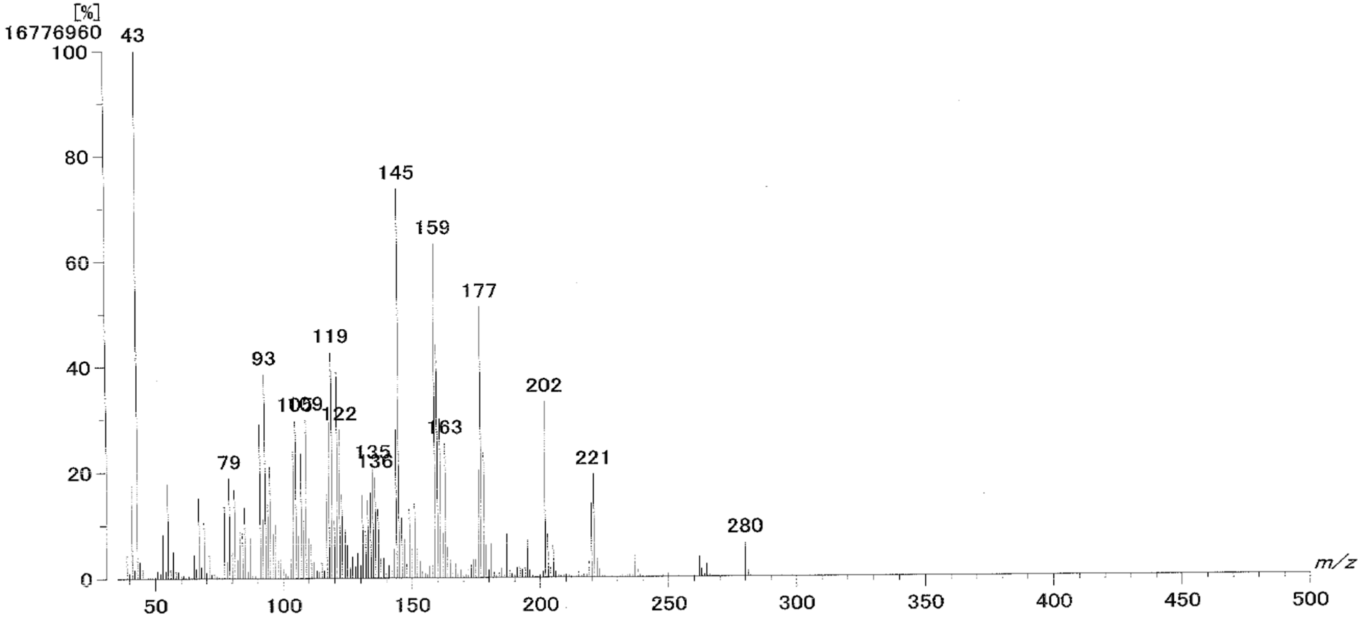

S43: LRCIMS EI-MS of **11**

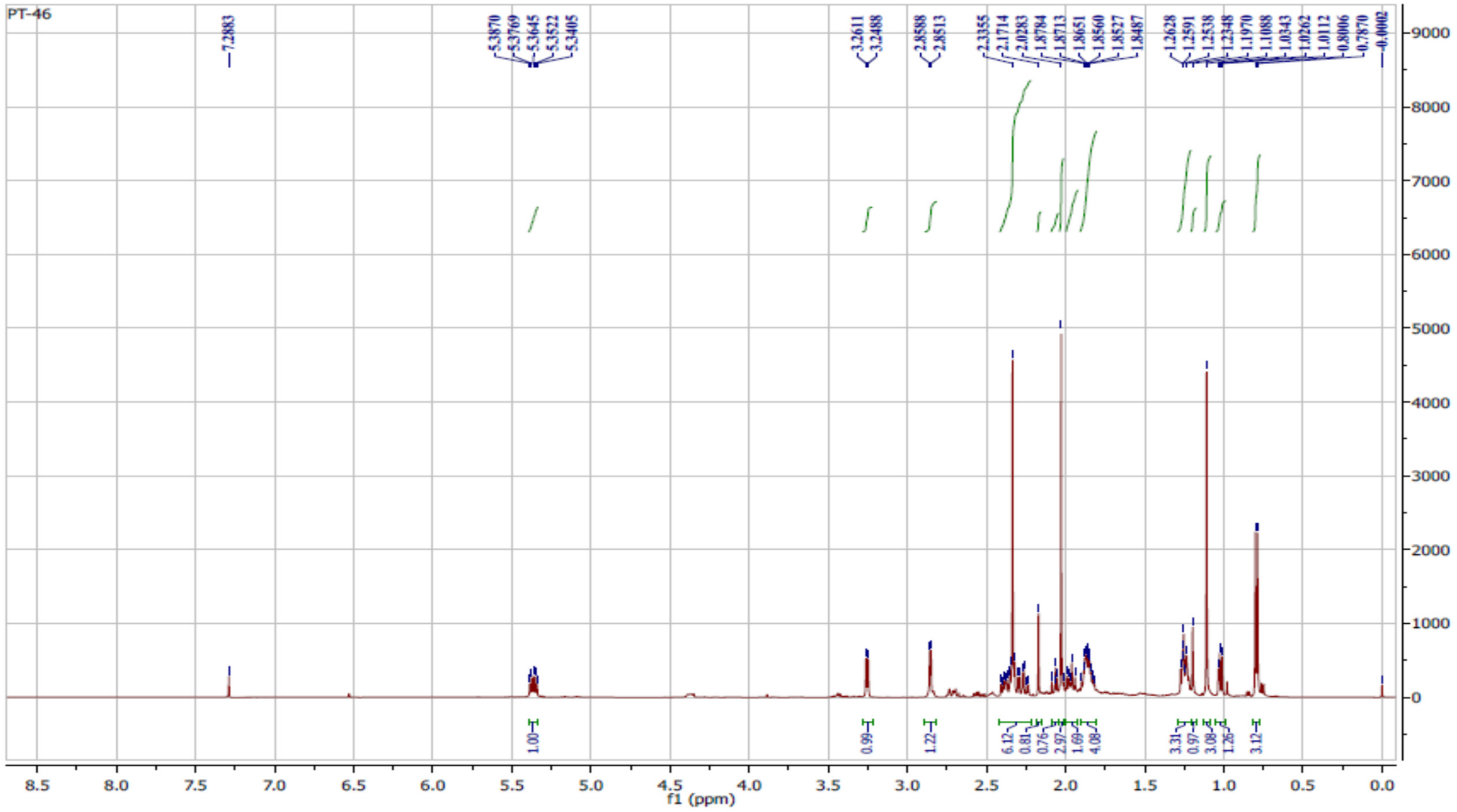

S44: ^{1}H-NMR of **11**

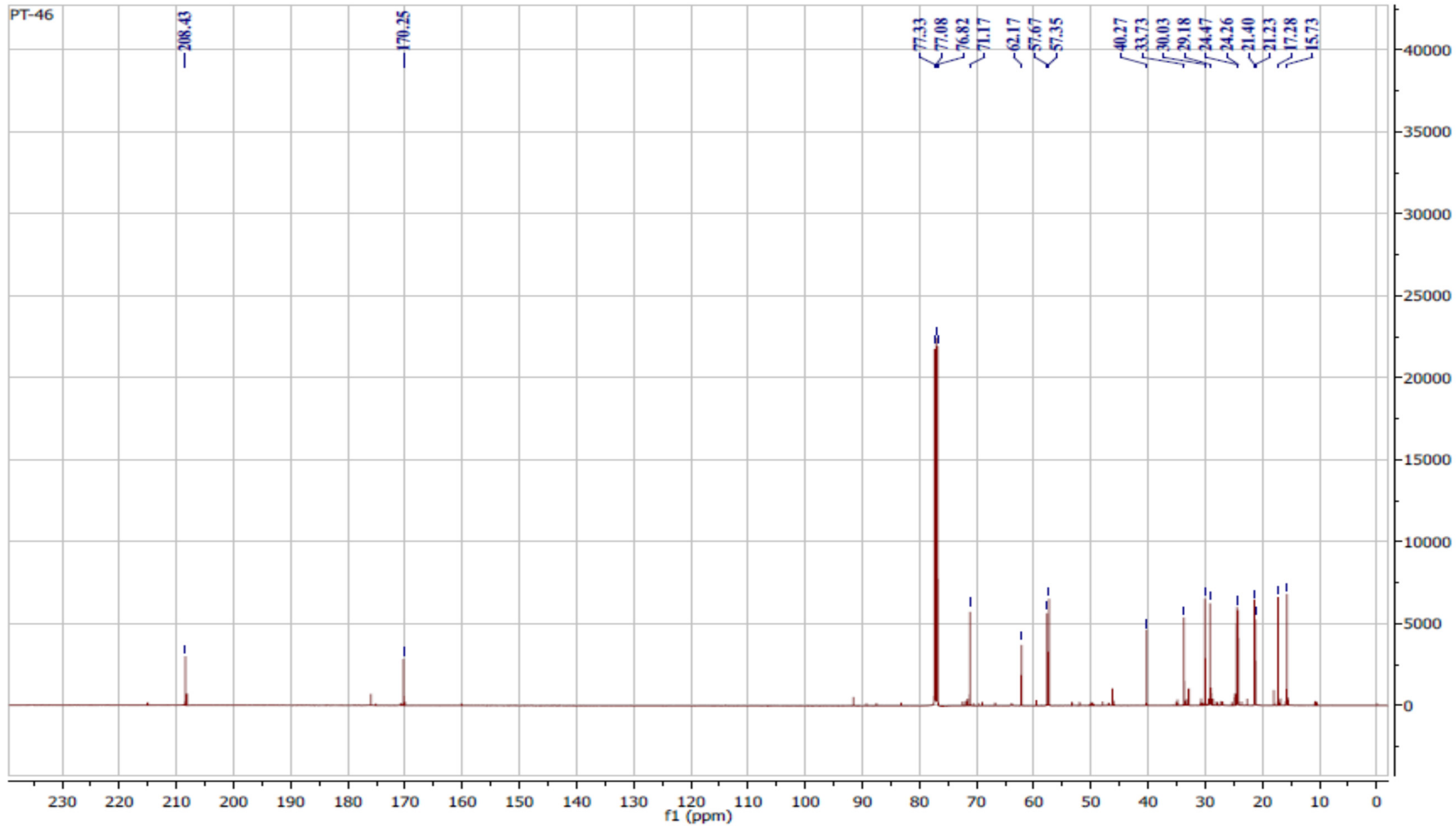

S45: ^{13}C-NMR of **11**

[Mass Spectrum]
Data : Umeyama-EI.17-Jul-2019.001 Date : 17-Jul-2019 10:25
Sample : PT-49
Note : MStation
Inlet : Direct Ion Mode : EI+
Spectrum Type : Normal Ion [MF-Linear]
RT : 0.67 min Scan# : 21
BP : m/z 177 Int. : 1444.04 (15141888)
Output m/z range : 35 to 500 Cut Level : 0.00 %

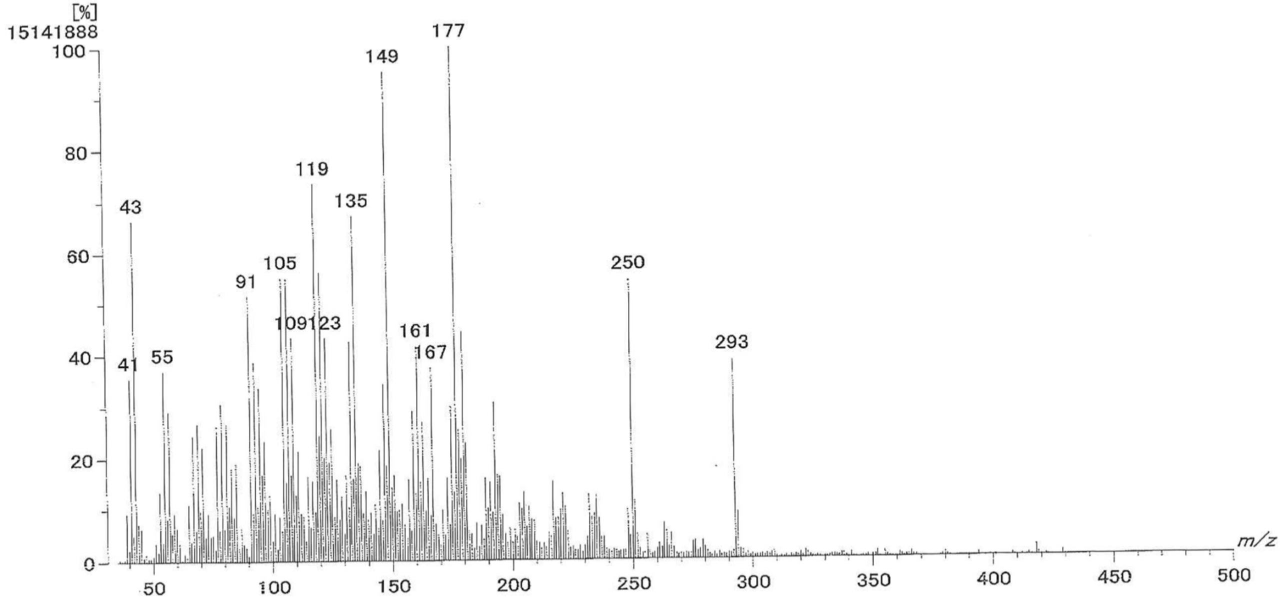

S46: LRCIMS EI-MS of **12**

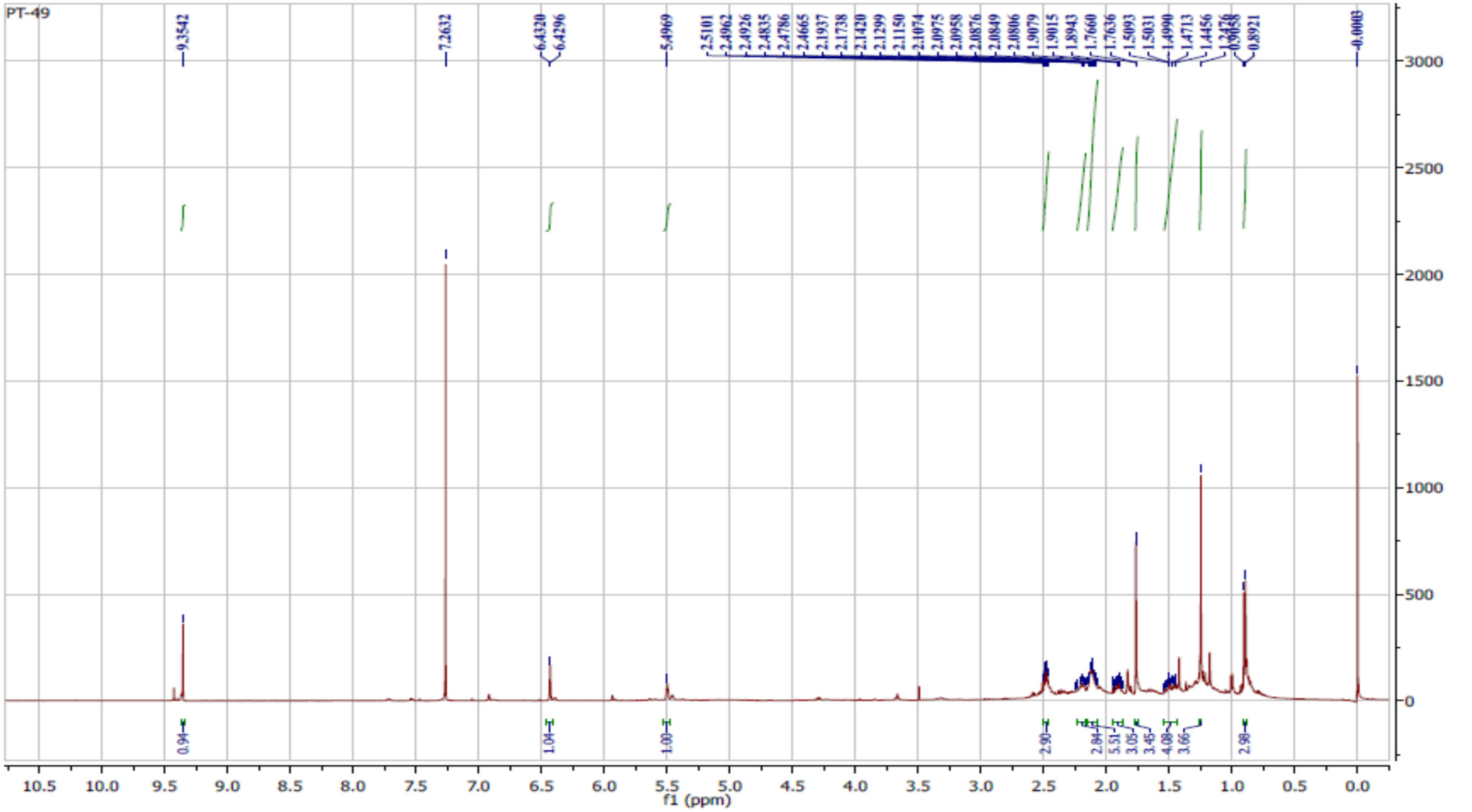

S47: ^{1}H-NMR of **12**

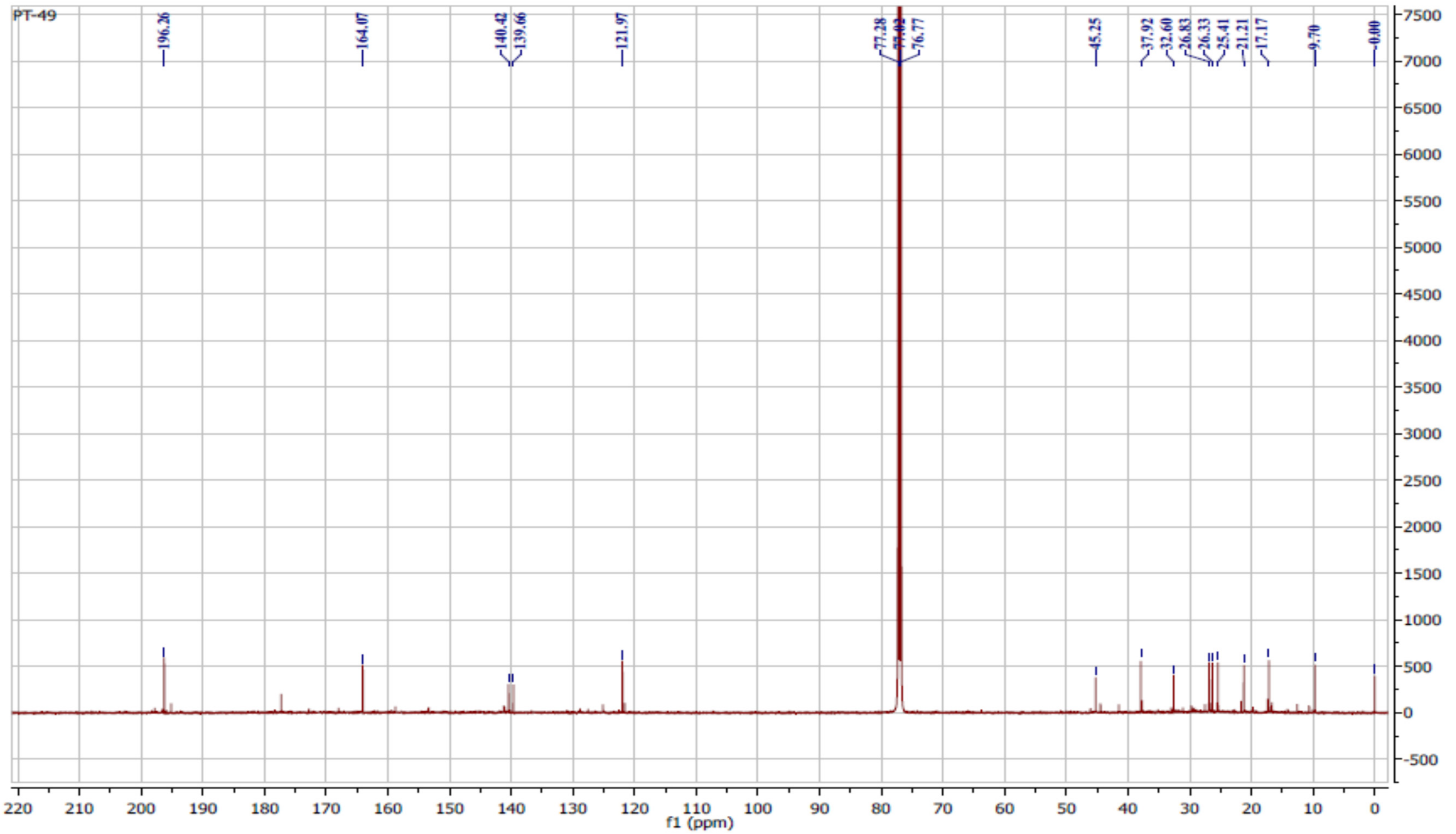

S48: ^{13}C-NMR of **12**
